# Organic J-Aggregate Nanodots with Enhanced Light Absorption and Near-Unity Fluorescence Quantum Yield

**DOI:** 10.1021/acs.nanolett.0c04928

**Published:** 2021-03-31

**Authors:** Hubert Piwoński, Shuho Nozue, Hiroyuki Fujita, Tsuyoshi Michinobu, Satoshi Habuchi

**Affiliations:** † King Abdullah University of Science and Technology, Biological and Environmental Science and Engineering Division, Thuwal 23955-6900, Saudi Arabia; ‡ Tokyo Institute of Technology, Department of Materials Science and Engineering, 2-12-1 O-okayama, Meguro-ku, Tokyo 152-8552, Japan

**Keywords:** Fluorescent nanoparticles, J-aggregates, enhanced light absorption, excited-state engineering, single-particle imaging, donor−acceptor−donor-type molecule

## Abstract

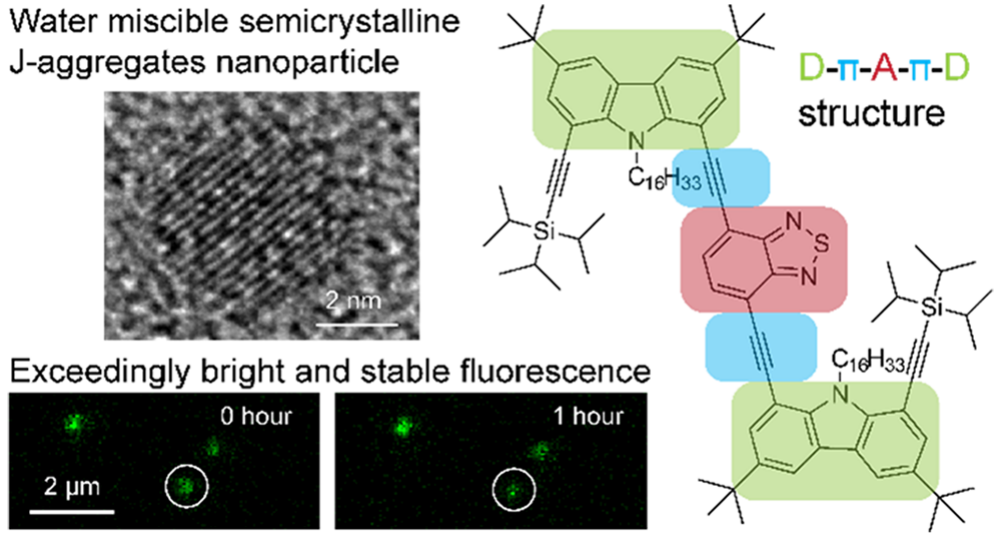

Development of biocompatible fluorophores with small size, bright fluorescence, and narrow spectrum translate directly into major advances in fluorescence imaging and related techniques. Here, we discover that a small donor–acceptor–donor-type organic molecule consisting of a carbazole (Cz) donor and benzothiazole (BT) acceptor (CzBTCz) assembles into quasi-crystalline J-aggregates upon a formation of ultrasmall nanoparticles. The 3.5 nm CzBTCz Jdots show a narrow absorption spectrum (fwhm = 27 nm), near-unity fluorescence quantum yield (ϕ_fl_ = 0.95), and enhanced peak molar extinction coefficient. The superior spectroscopic characteristics of the CzBTCz Jdots result in two orders of magnitude brighter photoluminescence of the Jdots compared with semiconductor quantum dots, which enables continuous single-Jdots imaging over a 1 h period. Comparison with structurally similar CzBT nanoparticles demonstrates a critical role played by the shape of CzBTCz on the formation of the Jdots. Our findings open an avenue for the development of a new class of fluorescent nanoparticles based on J-aggregates.

Advanced fluorescence imaging techniques have enabled visualization and characterization of biological structures and phenomena in unprecedented spatiotemporal resolution.^1^ Currently, development of new fluorescent probes is one of the major focus areas for the further advancement of state-of-the-art microscopy techniques.^2−5^ General characteristics of an ideal fluorescent probe include large fluorescence quantum yield (ϕ_fl_), large molar extinction coefficient (ε), large two-photon absorption cross section (δ), narrow spectral bandwidth, water solubility, bio- and photostability, low cytotoxicity, and small size. The last decade has seen a surge in the development of new fluorescent nanomaterials that include semiconductor quantum dots (Qdots),^6^ conjugated polymer nanoparticles (polymer dots),^7^ carbon dots,^8^ metal nanoclusters,^9^ lanthanide-doped nanoparticles,^10^ and dye-doped nanoparticles.^11^

Among others, organic molecules are a primary choice of the fluorophore in the vast majority of experimental settings. By harnessing their flexibility in the molecular design and synthesis, organic molecules that form nanoparticles with a bright fluorescence have been developed. A most frequently used strategy is to design molecules that maintain their fluorescence properties in an aggregate state by avoiding aggregate-induced fluorescence quenching. This has been achieved by either introducing a bulky group that prevents the molecules from π–π interaction^12^ or restricting intramolecular motion of conformationally flexible molecules that leads to aggregation-induced emission (AIE).^13,14^ Although these design concepts have been successful in the fabrication of highly fluorescent organic nanoparticles and used for imaging and sensing applications, a bright fluorescence has been achieved at the cost of high density of light-emitting moieties inside the particles as well as flexible molecular design, which is inevitable in these approaches.

An alternative and more attractive strategy is to modify fluorescence properties of organic nanoparticles through an aggregation-induced engineering of electronic states of the molecules inside the particles. A promising candidate for this approach is J-aggregates in which excited-state transition dipoles of monomer molecules in the aggregates are strongly coupled, leading to a delocalization of the excited state over a large number of monomers.^15−17^ This causes a significant change in spectroscopic properties of the molecules in the aggregates, including significant narrowing of both absorption and fluorescence spectra, enhancement of ε, preservation of (or even increased) ϕ_fl_, and decreased fluorescence lifetime (τ_fl_).^18^ Therefore, J-aggregates could, in principle, be an ideal fluorescent probe for fluorescence microscopy application. However, previously reported J-aggregates formed in an aqueous environment showed relatively low ϕ_fl_.^19−21^ Highly fluorescent J-aggregates have been reported only for a small number of molecules in a nonaqueous environment.^22^ Also, stable sub-5 nm J-aggregate nanoparticles have not been developed to date. These issues are originated mainly from the limited number of molecules that form J-aggregates.^23−26^

Here, we report sub-5 nm J-aggregate nanoparticles (Jdots) that display a near unity fluorescence quantum yield. Inspired by our previous study on ultrasmall donor–acceptor (D-π-*A*)-type polymer dots consisting of a carbazole (Cz) donor and benzothiadiazole (BT) acceptor that demonstrated polymer-chain-packing-dependent fluorescence properties,^27,28^ we conceived an idea of using the structural units of these polymers to fabricate nanoparticles with the expectation that we can better engineer photophysical characteristics of the molecules in an aggregation state. Surprisingly, we found that the fabricated nanoparticles exhibited the features of J-aggregates.

## Spectroscopic Characterization of CzBTCz Nanoparticles

**Figure 1:**
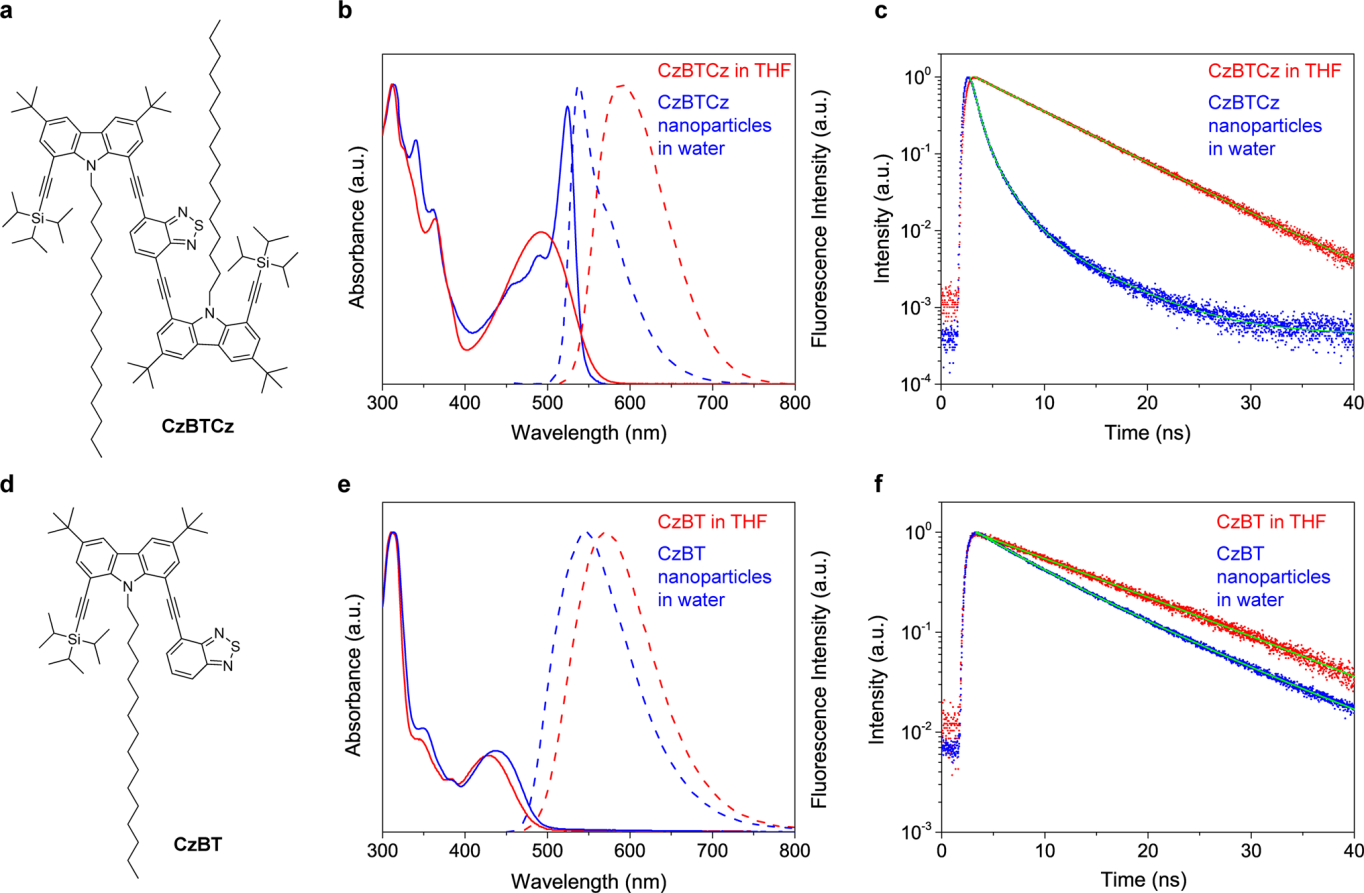
Spectroscopic properties of the CzBTCz Jdots. (a) Chemical structure of CzBTCz. (b) Steady-state absorption (solid lines) and fluorescence (dashed lines) spectra of CzBTCz in THF (red lines) and the CzBTCz nanoparticles dispersed in water (blue lines). The fluorescence spectra were measured upon excitation at 450 nm. (c) Fluorescence decays obtained for CzBTCz in THF (red) and the CzBTCz nanoparticles dispersed in water (blue). The solid lines show fitting of the data to single- or double-exponential decaying functions. (d) Chemical structure of CzBT. (e) Steady-state absorption (solid lines) and fluorescence (dashed lines) spectra of CzBT in THF (red lines) and the CzBT nanoparticles dispersed in water (blue lines). The fluorescence spectra were measured upon excitation at 420 nm. (f) Fluorescence decays obtained for CzBT in THF (red) and the CzBT nanoparticles dispersed in water (blue). The solid lines show fitting of the data to single- or double-exponential decaying functions.

We fabricated nanoparticles composed of CzBTCz (D-π-*A*-π-D structure, Figure 1a) and CzBT (D-π-*A* structure, Figure 1d) using the nanoprecipitation method (see the Materials and Methods in the Supporting Informaiton). CzBTCz in tetrahydrofuran (THF) showed broad featureless absorption (fwhm = 92 nm (3982 cm^–1^)) and fluorescence (fwhm = 97 nm (2690 cm^–1^)) spectra peaking at 492 and 590 nm, respectively, typical for charge-transfer (CT) absorption and fluorescence (Figure 1b, Table 1).^27^ The absorption band was previously attributed to intramolecular charge-transfer (ICT) absorption between the donor Cz and the acceptor BT moieties.^29^ CzBTCz in THF exhibited a high fluorescence quantum yield (ϕ_fl_ = 0.97) with a fluorescence lifetime of 6.5 ns (Figure 1c, Table 1).

**Table 1:** Spectroscopic Characteristics of CzBTCz and CzBT Nanoparticles

	ϕ_fl_	τ_fl_ (ns)	*k*_r_ (s^–1^)	*k*_nr_ (s^–1^)	ε (M^–1^ cm^–1^)	λ_abs_ (nm)	λ_fl_ (mn)	size (nm)^a^
CzBTCz^b^	0.97	6.5	1.5 × 10^8^	7.7 × 10^6^	2.4 × 10^4^	492	590	
CzBTCz Jdots^c^	0.95	1.7	5.7 × 10^8^	1.8 × 10^7^	8.1 × 10^6^	524	538	3.5
CzBT^b^	0.91	11.4	8.0 × 10^7^	7.9 × 10^6^	9.4 × 10^3^	428	570	
CzBT NPs^c^*^,^*^d^	0.32	8.7	3.7 × 10^7^	7.8 × 10^7^	3.2 × 10^6^	438	546	14
(CzBT)_*n*_ Pdots^c^*^,^*^e^	0.16	2.9	5.5 × 10^7^	2.9 × 10^8^	2.7 × 10^6^	497	631	3.0

a Diameter of the particles determined by TEM.

b Measured in THF.

c Measured in water.

d Nanoparticles (NPs).

e Polymer dots (Pdots).

In contrast to the frequently observed ICT spectra in THF, the fabricated CzBTCz nanoparticles dispersed in water displayed a distinctly narrower (fwhm = 27 nm (1036 cm^–1^)) and red-shifted absorption spectrum peaking at 524 nm (Figure 1b, Table 1). We also observed a significantly narrower fluorescence spectrum with vibronic structures peaking at 538 nm (fwhm = 55 nm (1800 cm^–1^), Figure 1b, Table 1) and a reduction of fluorescence lifetime (τ_fl_ = 1.7 ns, Figure 1c, Table 1) upon the particle formation (Supporting Note 1). The width of the fluorescence spectrum was much narrower than that in a nonpolar solution (Figure S1). Therefore, the observed spectral narrowing cannot be attributed to local environment-dependent spectral change.^29^ Interestingly, ϕ_fl_ remains almost unchanged (ϕ_fl_ = 0.95, Table 1).

The narrow and red-shifted absorption spectrum is a signature of strong coupling and high-order stacking of CzBTCz inside the nanoparticles.^15,16^ Further, the decrease in τ_fl_ observed for the CzBTCz nanoparticles is due to an increase in the radiative rate constant in the nanoparticles (*k*_r_^NPs^ = 5.7 × 10^8^ s^–1^) compared with that in THF (*k*_r_^THF^ = 1.5 × 10^8^ s^–1^) (Table 1). This enhanced radiative decay rate, a phenomenon usually referred to as exciton superradiance (see Supporting Note 2), is a key characteristic of J-aggregates.^30^ Together, our findings strongly suggest the formation of J-aggregates inside the CzBTCz nanoparticles (see Supporting Note 3). We emphasize that this study reports for the first time near unity ϕ_fl_ of J-aggregates in an aqueous environment.

The ε value of the CzBTCz nanoparticles was determined experimentally using fluorescence correlation spectroscopy (ε = 8.1 × 10^6^ M^–1^ cm^–1^_,_ see the Materials and Methods in the Supporting Information, Figure S2, and Supporting Note 4). This large ε value is interpreted at least partly by the theoretically predicted enhanced peak ε per monomer in strongly coupled J-aggregates (see Supporting Note 5).^31,32^ According to the theory, 3.6-fold enhancement of the peak ε is expected from the narrowing of the absorption spectra upon the particle formation. Such large enhancement of the peak ε is a result of the large exciton delocalization length (*N*_del_, number of molecules in an aggregate over which electron is delocalized). Indeed, *N*_del_ is calculated to be *N*_del_ = 19 using the change in the width of the absorption spectra^33^ upon the J-aggregates formation inside the particles (see Supporting Note 6). This value is much larger than that for previously reported water-miscible J-aggregate nanoparticles.^20^ The (CzBT)_*n*_ polymer dots^27^ whose monomer unit is identical to that of CzBTCz (Figure S3) but does not form J-aggregates inside the particles displayed three times smaller ε with similar particle size (see Table 1). This result further suggests the enhanced ε in the CzBTCz nanoparticles due to the intraparticle J-aggregates formation. In addition, the twist angle between the Cz and BT moiety inside the nanoparticles may contribute to the enhancement of the ε value (Supporting Note 7).

In contrast to CzBTCz, CzBT did not show any sign of J-aggregates upon the fabrication of the nanoparticles. The CT absorption band of the CzBT nanoparticles in water peaking at 437 nm showed a spectral width similar to that of CzBT in THF (Figure 1e). The CzBT nanoparticles in water also displayed a broad fluorescence spectra similar to those of CzBT in THF (fwhm_NPs_ = 109 nm (3567 cm^–1^), fwhm_THF_ = 113 nm (3385 cm^–1^), Figure 1e). We observed a slight decrease of the fluorescence lifetime of the CzBT nanoparticles (τ_fl_ = 8.7 ns) compared with that of CzBT in THF (τ_fl_ = 11.4 ns, Figure 1f). This reduction in τ_fl_ is mainly caused by an enhanced nonradiative decay rate *k*_nr_ (*k*_nr_^NPs^ = 7.8 × 10^7^ s^–1^, *k*_nr_^THF^ = 7.9 × 10^6^ s^–1^) rather than a change in *k*_r_ (see Table 1). The result suggests that the fluorescence of CzBT is quenched upon the nanoparticle formation. Indeed, we observed a significant reduction of ϕ_fl_ upon the particle formation (ϕ_fl_^NPs^ = 0.32, ϕ_fl_^THF^ = 0.91, see Table 1). Together, these results demonstrate that the aggregate-induced quenching occurs inside the CzBT nanoparticles, which is frequently observed in fluorescent organic nanoparticles.

The comparison between CzBT and CzBTCz indicates that the shape of the molecule plays a key role in the formation of the J-aggregates in the CzBTCz nanoparticles. The overall shape of a CzBTCz molecule is similar to those of cyanine dyes that often form J-aggregates.^34,35^ Cyanine dyes consist of two heterocyclic rings connected by a polymethine linker. Long aliphatic chains on the heterocyclic rings contribute to efficient formation of J-aggregates. CzBTCz consists of two Cz moieties with long alkyl chains bridged by triple bonds with a BT moiety. Our findings strongly suggest that the unique spatial arrangement of the Cz donor and BT acceptor in the CzBTCz molecule enables the efficient formation of the J-aggregates in the nanoparticles. We would like to point out that unlike the CzBTCz nanoparticles, J-aggregates of cyanine dyes show much lower ϕ_fl_,^36−38^ highlighting the uniqueness of highly fluorescent CzBTCz J-aggregate nanoparticles (Supporting Note 8).

## Characterization of Semicrystalline Structure of CzBTCz Jdots

**Figure 2:**
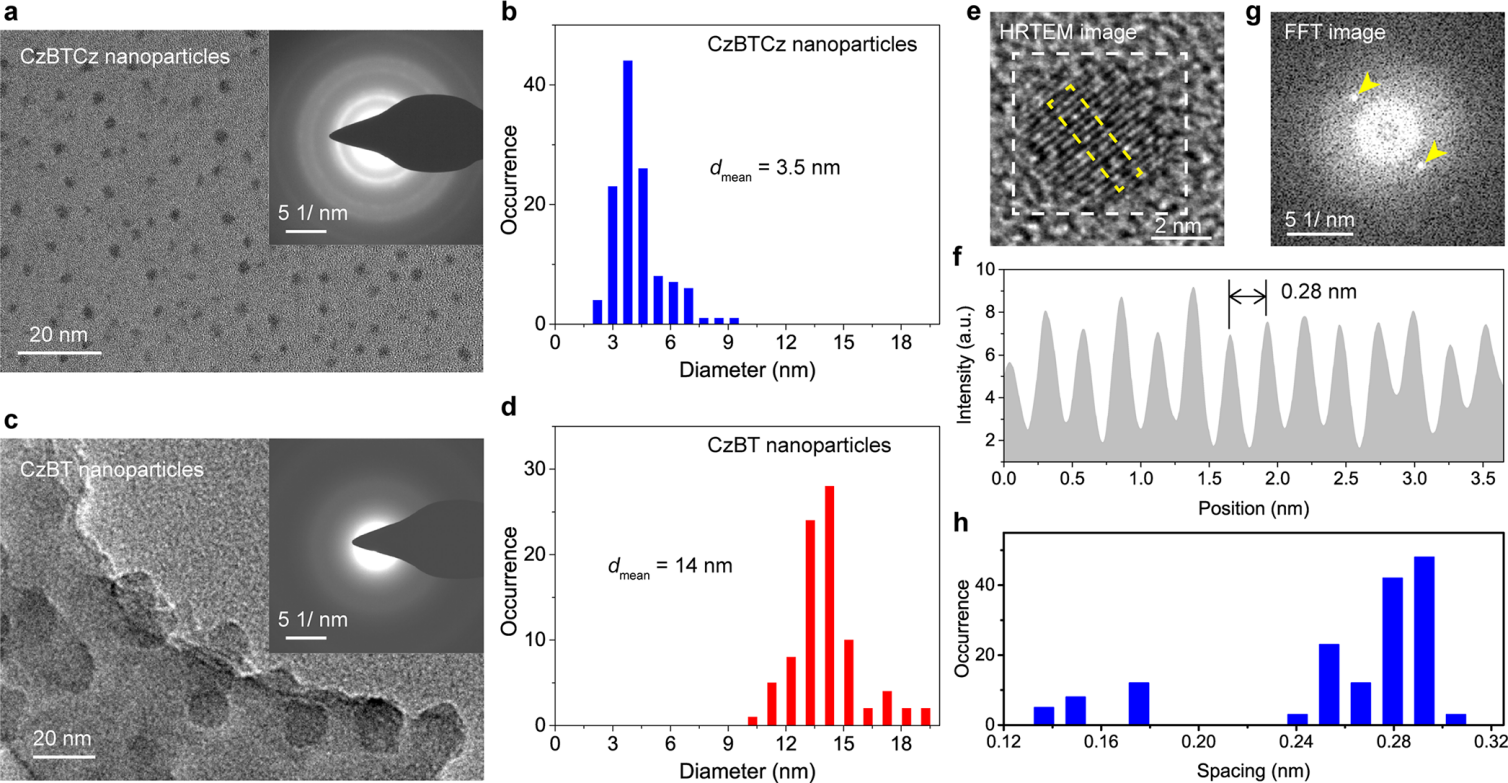
Structural characterization of the CzBTCz Jdots. (a) Transmission electron microscopy (TEM) image of the CzBTCz nanoparticles. The inset shows a selected area electron diffraction (SAED) pattern obtained from the particles. (b) Frequency histogram showing the size distribution of the CzBTCz nanoparticles. (c) TEM image of the CzBT nanoparticles. The inset shows a SAED pattern obtained from the particles. (d) Frequency histogram showing the size distribution of the CzBT nanoparticles. (e) High-resolution TEM image of a single CzBTCz nanoparticle. The yellow box shows the region from which the intensity profile displayed in (f) was calculated. The white box shows the region from which the fast Fourier transform (FFT) image displayed in (g) was calculated. The yellow arrows in (g) highlight spots in the FFT image that confirm the presence of the single-crystalline-like periodical structure in the CzBTCz nanoparticle displayed in (e). (h) Frequency histogram showing the distribution of the spacing observed in the CzBTCz nanoparticles.

We next characterized structural properties of the CzBTCz nanoparticles. A transmission electron microscopy (TEM) image of the CzBTCz nanoparticles showed the formation of spherical-shaped nanoparticles with a relatively narrow size distribution with a mean diameter of 3.5 nm (Figure 2a and 2b, Supporting Note 9). A selected area electron diffraction (SAED) pattern obtained from the CzBTCz nanoparticles clearly showed sharp rings, a typical spatial pattern observed in polycrystalline materials (Figure 2a inset). In contrast, the crystalline structure was not observed for the CzBT nanoparticles that have a spherical shape with a mean diameter of 14 nm (Figure 2c, 2d). Instead, we observed a halo-like SAED pattern from the CzBT nanoparticles, a typical spatial pattern observed from amorphous materials (Figure 2c inset). A high-resolution (HR) TEM image of the CzBTCz nanoparticles clearly revealed lattice fringes with a 0.28 nm lattice spacing (Figure 2e, 2f). A fast Fourier transform (FFT) analysis of the image also indicates a lattice spacing of 0.28 nm (Figure 2g, 2h). While most of the 3.5 nm CzBTCz nanoparticles showed a single crystalline domain, (Figure 2e, 2f, 2g), a small fraction of the nanoparticles, in particular larger nanoparticles, exhibited multicrystalline domains inside the particles (Figure S4).

These findings are consistent with the spectroscopic properties of the CzBTCz and CzBT nanoparticles and strongly suggest the formation of ultrasmall 3.5 nm CzBTCz Jdots in which the CzBTCz molecules form strongly coupled J-aggregates. The 0.28 nm lattice fringe observed for the CzBTCz Jdots is similar to the previously reported spatial periodicity of other organic J-aggregates (0.26–0.31 nm).^39,40^ In addition, the 0.28 nm spacing is smaller than the distance between π–π stacked (i.e., weak coupling) triisopropylsilylethynyl pentacene molecules (0.32–0.35 nm), which has structural similarity with CzBTCz (i.e., flat aromatic ring connected to triisopropylsilyl groups through triple bonds).^41^ These results further suggest that strongly coupled J-aggregates are formed inside the CzBTCz Jdots. We note that we cannot rule out the possibility that graphene-like carbon nanoparticles are generated by the electron beam irradiation during the HRTEM experiments as the lattice parameters of graphene (0.21, 0.24, and 0.34 nm) are close to the observed 0.28 nm lattice spacing (Supporting Note 10).^42^

An origin of the observed size difference between the CzBTCz Jdots and the CzBT nanoparticles is not clear at the moment. The large zeta potential (−45 mV) of the CzBTCz Jdots compared with that of the CzBT nanoparticles (−12 mV) indicates better colloidal stability of the CzBTCz Jdots. This higher colloidal stability may contribute to the formation of the ultrasmall 3.5 nm size Jdots without further growth or nonspecific aggregations of the particles.

## Two-Photon Absorption of CzBTCz Jdots

**Figure 3:**
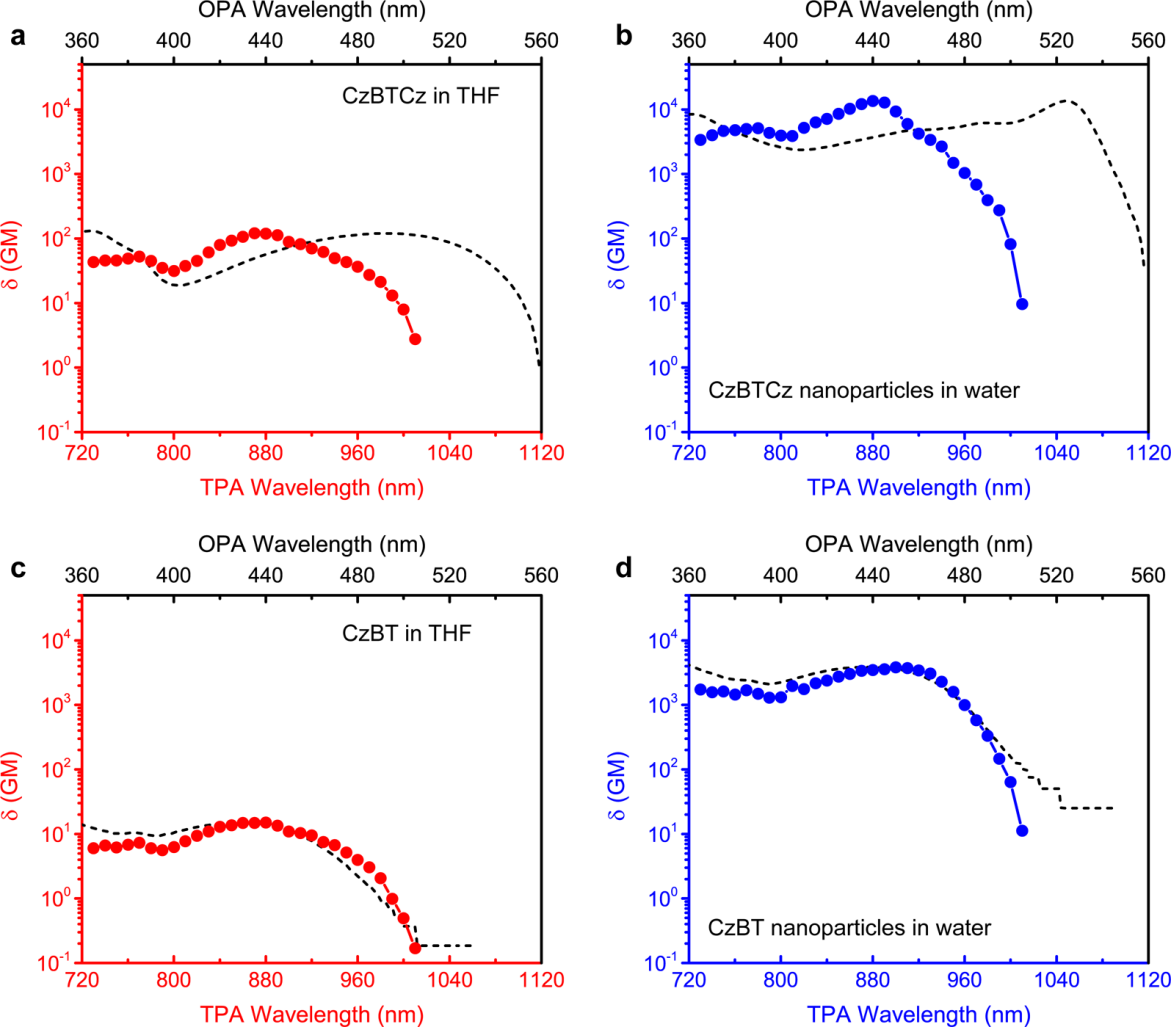
Two-photon absorption properties of the CzBTCz Jdots. (a) Two-photon absorption spectrum of CzBTCz in THF. The dashed line shows a one-photon absorption spectrum of CzBTCz in THF. (b) Two-photon absorption spectrum of the CzBTCz nanoparticles dispersed in water. The dashed line shows a one-photon absorption spectrum of the CzBTCz nanoparticles dispersed in water. (c) Two-photon absorption spectrum of CzBT in THF. The dashed line shows a one-photon absorption spectrum of CzBT in THF. (d) Two-photon absorption spectrum of the CzBT nanoparticles dispersed in water. The dashed line shows a one-photon absorption spectrum of the CzBT nanoparticles dispersed in water.

We next investigated two-photon absorption (TPA)-induced fluorescence of the CzBTCz Jdots (Figures S5 and S6). CzBTCz in THF showed a relatively large TPA cross-section (δ = 120 GM, Figure 3a), which is 8 times larger than that of CzBT in THF (δ = 15 GM, Figure 3c). The large TPA cross-section of CzBTCz can be attributed to its symmetric D-π-*A*-π-D structure, which has been known as one of the best structures for a large TPA cross-section.^43,44^ The TPA spectrum of CzBTCz in THF showed a significant blue shift compared with a one-photon absorption (OPA) spectrum of CzBTCz in THF (Figure 3a, Figure S7), which can be attributed to different selection rules for OPA and TPA in quadrupole molecules.^43,45^ In contrast, CzBT in THF showed similar OPA and TPA spectra (Figure 3c, Figure S7), which is expected for an asymmetric D-π-*A* type molecule.

The CzBTCz Jdots dispersed in water displayed a narrow TPA spectrum (fwhm = 32 nm, Figure 3b, Figure S7). The width of the spectrum is similar to the OPA spectrum of the CzBTCz Jdots (fwhm = 27 nm), indicating that the spectroscopic unit of the J-aggregates in the particle that are responsible for the TPA is similar to that in the OPA. However, we observed significant blue shift of the TPA spectrum of the CzBTCz Jdots compared with the OPA spectrum of the CzBTCz Jdots (Figure 3b, Figure S7). Indeed, the peak wavelength of the TPA spectrum of the CzBTCz Jdots is similar to that of CzBTCz in THF (Figure 3a and 3b, Figure S7). This result suggests that the selection rule for the TPA of the CzBTCz is not affected significantly by the J-aggregates formation inside the particles. Due to the high packing density of the CzBTCz molecules in the Jdots, we observed a very large TPA cross-section of the 3.5 nm size CzBTCz J-dots (δ = 13,540 GM, Figure 3b). The TPA spectrum of the CzBT nanoparticles is very similar to that of CzBT in THF (Figure 3D), which is consistent with the results obtained from the OPA spectra and demonstrated the absence of specific intermolecular interaction inside the particles.

## Fluorescence Brightness of CzBTCz Jdots

**Figure 4:**
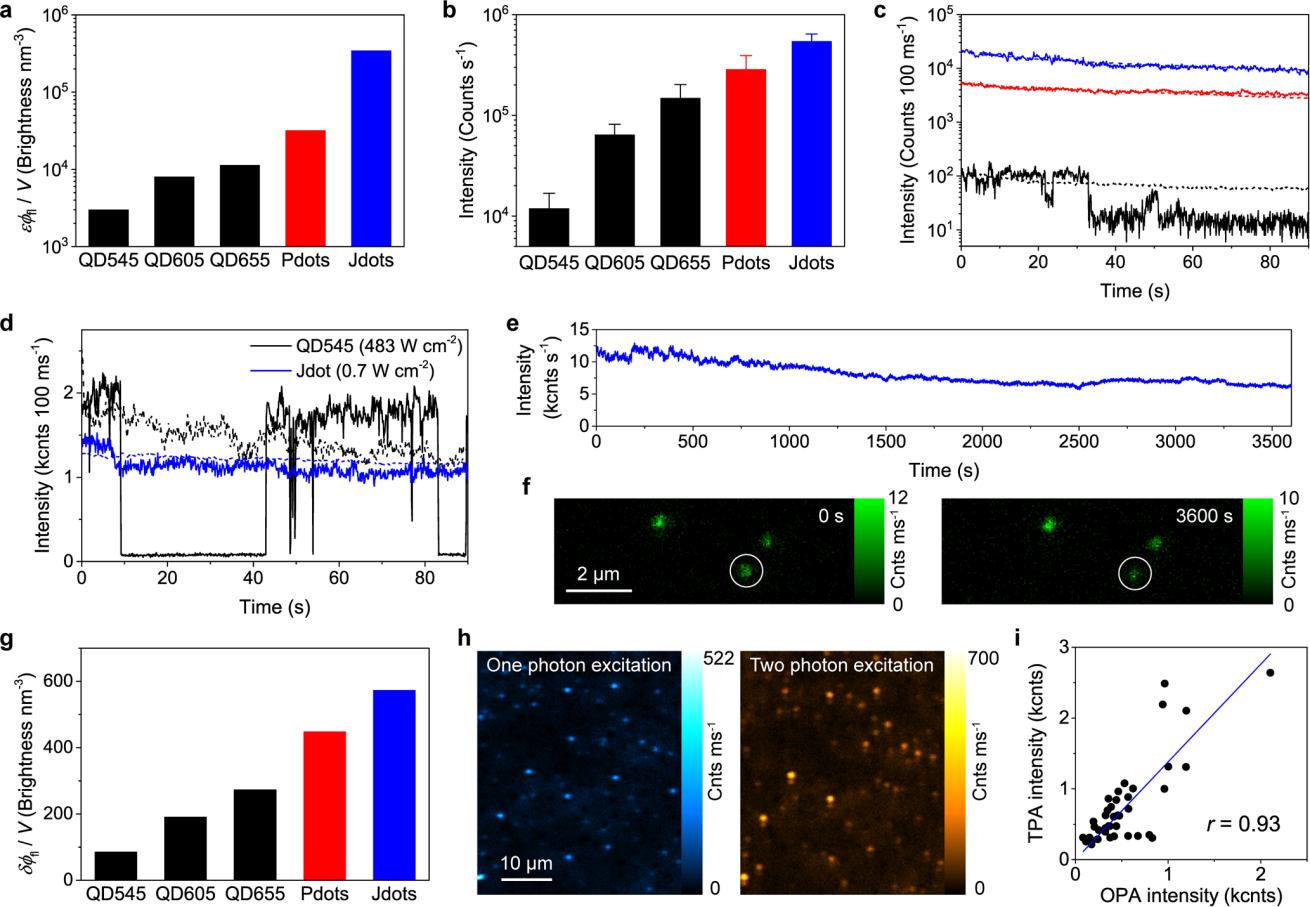
Fluorescence brightness of the CzBTCz Jdots. (a) Fluorescence brightness per unit volume calculated for Qdots 545 (QD545), Qdots 605 (QD605), Qdots 655 (QD655), (CzBT)_*n*_ polymer dots (Pdots), and CzBTCz Jdots (Jdots) under the one-photon excitation condition. (b) Mean fluorescence intensities of individual QD545, QD605, QD655, Pdots, and Jdots obtained under identical experimental conditions (485 nm excitation, 424 W cm^–2^ excitation power). Error bars show standard deviations determined by 1275, 586, 1663, 611, and 1871 molecules for the QD545, QD605, QD655, Pdots, and Jdots, respectively. (c) Examples of fluorescence intensity trajectories of single Qdots 545 (black line), (CzBT)_*n*_ Pdots (red line), and CzBTCz Jdots (blue line) obtained under identical experimental conditions (485 nm excitation, 160 W cm^–2^ excitation power). The dashed lines show mean fluorescence intensity trajectories of 65, 84, and 115 molecules for the Qdots 545, (CzBT)_*n*_ Pdots, and CzBTCz Jdots, respectively. (d) Examples of fluorescence intensity trajectories of single Qdots 545 (black line) and CzBTCz Jdots (blue line) obtained under conditions that gave similar fluorescence intensity (488 nm excitation, 483 and 0.7 W cm^–2^ excitation power for the Qdots 545 and CzBTCz Jdots). The dashed lines show mean fluorescence intensity trajectories of 108 and 60 molecules for the Qdots 545 and CzBTCz Jdots, respectively. (e) Fluorescence intensity trajectory obtained from a single CzBTCz Jdot over a 1 h period at the excitation power of 0.7 W cm^–2^. (f) Fluorescence images of the CzBTCz Jdots captured at time point 0 s (left) and 3600 s (right). White circles highlight the Jdot from which the intensity time trajectory shown in (e) was obtained. (g) Fluorescence brightness per unit volume calculated for Qdots 545, Qdots 605, Qdots 655, (CzBT)_*n*_ Pdots, and CzBTCz Jdots under the two-photon excitation condition. (h) Fluorescence images of single CzBTCz Jdots captured under one-photon excitation (left, 488 nm excitation, 27 W cm^–2^ excitation power) and two-photon excitation (right, 880 nm excitation, 1.85 kW cm^–2^ excitation power) conditions. (i) Fluorescence intensity obtained from individual CzBTCz Jdots under one- and two-photon excitation conditions. Each dot shows intensities obtained for a single Jdot by one- or two-photon excitation. The solid line shows a linear fit, and *r* is Pearson’s correlation coefficient.

The results reported in this study demonstrated that the CzBTCz Jdots have excellent characteristics as a fluorescent probe, including the narrow absorption bandwidth (fwhm = 27 nm), J-aggregation-induced enhanced peak ε (ε = 8.1 × 10^6^ M^–1^ cm^–1^) and δ (δ = 13,540 GM), near unity ϕ_fl_ (ϕ_fl_ = 0.95), ultrasmall particle size (*d* = 3.5 nm), and high stability in aqueous solution. The relationship *εϕ*_fl_/*V* (i.e., fluorescence brightness per unit volume) provides a benchmark for overall brightness of fluorescent probes, where *V* is the volume of the probe. A calculated *εϕ*_fl_/*V* value of the CzBTCz Jdots (3.4 × 10^5^) is two orders of magnitude larger than that determined for semiconductor quantum dots (Qdots) that emit photoluminescence in the similar wavelength region (Figure 4a, Table S1, Figure S8), mainly due to its very large ε and very small size. Further, the *εϕ*_fl_/*V* value is an order of magnitude larger than that of the (CzBT)_*n*_ polymer dots that consist of the polymer with the same repeating unit as CzBTCz (Figure 4a). The near unity ϕ_fl_ of CzBTCz under the densely packed conditions in the particles enables the exceedingly high *εϕ*_fl_/*V* value (Supporting Note 11).

A comparison of fluorescence intensity trajectories obtained from single CzBTCz Jdots, (CzBT)_*n*_ polymer dots, and Qdots545 at an identical data acquisition condition indeed revealed that the CzBTCz Jdots show much brighter fluorescence than the (CzBT)_*n*_ polymer dots and Qdots 545 (Figure 4b and 4c, Supporting Note 11). The very large peak ε due to the presence of multiple emitters inside the particles is responsible for the brighter fluorescence of the Jdots compared with the Qdots that have only a single emitter inside the particles. Further, fluorescence intensity trajectories of these fluorophores recorded at different excitation powers that result in a similar photon count rate, which is more relevant to fluorescence imaging applications, demonstrated that the CzBTCz Jdots exhibited stable fluorescence over a long period of time without displaying any fluorescence blinking behavior (Figure 4d, 4e). Even after 1 h of continued excitation, we were able to detect bright fluorescence from a single CzBTCz Jdots (Figure 4e, 4f). The CzBTCz Jdots also exhibited higher fluorescence brightness per unit volume under the two-photon excitation condition (*δϕ*_fl_/*V*) compared with the Qdots and polymer dots (Figure 4g, Table S1). Due to the very large *δϕ*_fl_/*V* value, two-photon excitation fluorescence of individual CzBTCz Jdots was easily detected with less than 2 kW cm^–2^ excitation power (Figure 4h, Figure S9). The fluorescence brightness of the particles under one- and two-photon excitation conditions showed a linear relationship (Figure 4i), implying that both the ε and δ of each CzBTCz Jdot are determined simply by the particle size. These results together with its ultrasmall size collectively highlight the superior characteristics of the CzBTCz Jdots as a fluorescent probe, in particular in single-molecule level fluorescence imaging applications.

## Conclusion

In summary, we developed small-molecule-based highly fluorescent organic nanoparticles with unique optical properties using a reprecipitation method. The periodical pattern found in the CzBTCz Jdots demonstrates that the molecules are packed in a spatially ordered manner, consistent with the spectroscopic data that suggest the formation of J-aggregates. The absence of the J-aggregates formation in the CzBT nanoparticles consisting of the same backbone but different shape than CzBTCz illustrates a critical role played by the shape of the molecule on their spatial packing inside the particles. Our result suggests that the structure of CzBTCz, which mimics the generic cyanine dye structure consisting of two large heterocyclic components connected by a π-conjugated linker, is highly relevant to the J-aggregate formation. The heavy-metal-free organic Jdots constitute a promising biocompatible alternative to semiconductor nanomaterials for one- and two-photon fluorescence imaging applications. Careful design and synthesis of carbazole-based symmetric D-π-*A*-π-D molecules with different acceptor moieties could enable researchers to tune the fluorescence into the near-infrared (NIR) and shortwave infrared (SWIR) spectral regions,^28,46^ which is more desirable in biological imaging. Utilization of self-assembled J-aggregates nanostructures could solve the common problem of limited brightness of NIR/SWIR-emitting fluorescent probes.

## Supporting Information Available

Materials and methods, supporting notes S1–S11, Figures S1–S10, and Table S1 (PDF)
