# Supplementary material for: Organic J-Aggregate Nanodots with Enhanced Light Absorption and Near-Unity Fluorescence Quantum Yield
Source: Nano Lett. 2021 Mar 31;21(7):2840–7. doi: 10.1021/acs.nanolett.0c04928 (PMC8155199; doi:10.1021/acs.nanolett.0c04928)
Supplement: Supplementary file 1 — nl0c04928_si_001.pdf [file nl0c04928_si_001.pdf]

Supporting Information for

Organic J-aggregate Nanodots with Enhanced Light Absorption and Near-Unity Fluorescence Quantum Yield

Hubert Piwoński,[†*] *Shuho Nozue,*[†] *Hiroyuki Fujita,*[‡] *Tsuyoshi Michinobu,*[‡] *Satoshi Habuchi*[†*]

†King Abdullah University of Science and Technology, Biological and Environmental Science and Engineering Division, Thuwal 23955-6900, Saudi Arabia

‡Tokyo Institute of Technology, Department of Materials Science and Engineering, 2-12-1 O-okayama, Meguro-ku, Tokyo 152-8552, Japan

*Correspondence should be addressed to S.H. (E-mail: Satoshi.Habuchi@kaust.edu.sa) or H.P. (E-mail: Hubert.piwonski@kaust.edu.sa)

Contents

Materials and Methods

Supporting Notes 1-11

Supporting Figures: Figures S1–S10

Supporting Table: Table S1

Materials and Methods

Preparation of the organic nanoparticles. Procedures for the synthesis of CzBT and CzBTCz are described elsewhere.[1] Semiconductor quantum dots, Qdot 545, Qdot 605, and Qdot 655 ITK carboxyl quantum dots, and TetraSpec fluorescent microspheres (0.1 μm diameter) were purchased from Invitrogen (ThermoFisher Scientific, Invitrogen). Fluorescent polystyrene microspheres doped with dragon green dye (50 nm diameter) were purchased from Bangs Laboratories. Rhodamine 6G was purchased from Acros Organics. THF was purchased from Sigma-Aldrich. Organic nanoparticles were fabricated by a modified reprecipitation method. Stock solutions were prepared by dissolving 1 mg of CzBT or CzBTCz in 10 ml of fresh THF, briefly sonicated in an ultrasonic bath (37 kHz, Elma Schmidbauer GmbH, Elmasonic P60H), and filtered through a 0.1-μm PTFE membrane filter (Whatman, Puradisc 6784-1301). A 165 μl of the solution was then rapidly injected into 12 ml of water in a glass vial, and the mixed solution was kept under sonication in an ultrasonic bath at 277 K for 30 min. THF in the mixed solution was removed by keeping the solution under the vacuum for 1 hour, followed by a continuous flow of compressed dry air to concentrate the colloidal suspension. The resulting nanoparticles' suspension was further filtered through a 0.1-μm PTFE membrane filter. The fabrication of the $(CzBT)_n$ polymer dots was described elsewhere.[2]

Functionalization of glass coverslip. Glass coverslips were cleaned by 15 min sonication in ethanol (24106, Sigma-Aldrich) followed by 15 min sonication in 1M KOH and rinsed thoroughly with MilliQ water (Millipore, Milli-Q Reference) and repeated 15 min sonication in ethanol. Prior to surface functionalization any traces of water were removed by three times repeated rinsing and 15 min sonicating of coverslip in acetone (20067.320, VWR Chemicals) which was finally followed by immersion in 2% (v/v) (3-aminopropyl)-triethoxysilane (A3648, Sigma-Aldrich) in

acetone. After 2 minutes of incubation, the coverslips were thoroughly rinsed in excess of MilliQ water, dried and baked for 30 minutes at 110 °C in an oven. The samples for all the single-particle measurements were prepared by depositing colloidal solutions of the nanoparticles on silanized surface of clean coverslips. The samples for the single-molecule measurements on the organic dye, rhodamine 6G (R6G), were prepared by spin coating the toluene solution containing R6G and polymethyl methacrylate (Acros Organics) on clean coverslips.

Structural characterization of the nanoparticles. The zeta potential of the nanoparticles was measured on a dynamic light scattering Malvern Zetasizer NanoZS. TEM micrographs and selected area electron diffraction patterns of the nanoparticles were obtained using Titan 80-300 kV CT (FEI) electron microscope. The TEM grids were treated with plasma before sample preparation. The samples for the TEM measurements were prepared by drop-casting colloidal solutions of the nanoparticles onto a holey carbon film on Ni grids (EMS, FCN-100NI) or Cu grids (EMS, Q225-CMA) and drying them under vacuum by keeping in desiccator for 1 hour before the measurement.

Ensemble spectroscopic characterization. Steady-state absorption and fluorescence measurements were performed in a U-3900 Spectrophotometer (Hitachi High-Technologies) to collect absorption spectra and in a Fluoromax-4 spectrofluorometer (Horiba Scientific) to collect fluorescence spectra. Fluorescence spectra were corrected using the spectral response of the photodetector.

The fluorescence quantum yield (ϕ_{fl}) was determined using rhodamine 6G (Acros Organics, ϕ_{fl} = 0.95; ethanol) and fluorescein (Acros Organics, ϕ_{fl} = 0.95; 0.1M NaOH) as reference standards.

ϕ_{fl} was calculated from the gradient of linear relation between absorbance and integrated fluorescence intensities recorded for different sample dilutions, according to equation 1:

$$\phi_{\mathrm{X}} = \phi_{\mathrm{ST}} \left(\frac{Grad_{\mathrm{X}}}{Grad_{\mathrm{ST}}}\right) \left(\frac{\eta_{\mathrm{X}}^2}{\eta_{\mathrm{ST}}^2}\right) \qquad (1)$$

where the subscripts ST and X denote standard and tested sample, respectively, and ϕ is the fluorescence quantum yield. *Grad* is the gradient from the plot of integrated fluorescence intensity vs absorbance, and η is the refractive index of the solvent.

Fluorescence decays were measured using a time-correlated single-photon counting (TCSPC) setup integrated in a home-built scanning confocal microscope (see below).[2] Samples were excited at 485 nm using an LDH-D-C-485 picosecond pulsed diode laser (PicoQuant, pulse width ≈ 100 ps, repetition = 20 MHz). Fluorescence from the samples was detected using a single photon avalanche photodiode (SPAD, PicoQuant, τ-SPAD-50). The TCSPC measurements were conducted using a TCSPC module (PicoQuant, HydraHarp 400). The module was controlled by SymPhoTime64 software (PicoQuant). The fluorescence decay curves were fitted to either single- or double-exponential decaying functions using FluoFit software (PicoQuant).

Determination of two-photon absorption cross-section of the nanoparticles. Two-photon absorption (TPA) of the samples was characterized using a wavelength-tunable femtosecond pulsed Ti:sapphire laser (pulse width = 140 fs, repetition rate = 80 MHz, Coherent, Chameleon Ultra) as an excitation light source. The laser output beam was expanded by a 5× beam expander (Thorlabs, GBE05-B) and attenuated by a variable iris diaphragm to approximately 0.5 cm in diameter. The intensity of the laser beam was controlled by rotating a half-wave plate (Thorlabs; AHWP10M-980) mounted at the front of a laser-Glan polarizer (Thorlabs, GL10-B). A linearly polarized light was used in all the experiments. The beam intensity was monitored by a digital

power meter (Thorlabs, PM100D) equipped with thermal power sensors (Thorlabs, S470C). The excitation laser beam was focused with an achromatic cylindrical lens (50 mm focal length) onto a 2 mm thick quartz cuvette (Thorlabs, CV10Q700) mounted in a cuvette holder (Thorlabs, CVH100) equipped with a band-pass filter (Semrock, FF01-609/181), collimating optic and fiber adapter. The fluorescence was collected at an angle perpendicular to the direction of the excitation beam by a multimode optical fiber (Thorlabs, BFL200LS02), which is connected to a spectrograph (Andor Technology, SR-303i-B) equipped with an EMCCD camera (Andor Technology, Newton 970P-BV). In order to measure two-photon absorption fluorescence excitation spectra, fluorescence spectra were recorded upon excitation with the pulsed laser at wavelength region between 720 – 1010 nm at every 10 nm. Each spectrum was measured with 5 s integration time. Integrated fluorescence intensities were plotted as a function of the excitation wavelength to obtain two-photon excitation spectra.

TPA cross-sections (δ) of the samples were determined with respect to a standard dye, rhodamine B (Acros Organics) whose δ is known. The δ values were calculated by the equation 2[3]:

$$\delta_S = \delta_R\left({}^{F_S \cdot \phi_R \cdot C_R \cdot n_S}/_{F_R \cdot \phi_S \cdot C_S \cdot n_R}\right) \qquad (2)$$

where, δ_R is TPA cross-section of rhodamine B in ethanol used as a reference, F is the integrated area of the measured two-photon TPA-induced fluorescence spectrum of the sample (F_S) and reference (F_R), ϕ is the fluorescence quantum yields of the sample (ϕ_S) and reference (ϕ_R), C is the concentration of the sample (C_S) and reference (C_R) and n is the refractive indexes of the solvents used for the sample (n_S) and reference (n_R). We assumed that the one-photon and the two-photon fluorescence quantum efficiencies are equal and constant over the spectral range of the experiment,

as the spectra of two-photon-absorption-induced fluorescence are nearly identical to that of the one-photon excited fluorescence (Figure S7) and originate from the S_1 state no matter how many photons are simultaneously absorbed for a single molecular transition.[4]

Single-particle confocal fluorescence microscopy. Single-particle fluorescence measurements were conducted on a home-built scanning confocal microscope based on an Olympus IX 71 platform[2] equipped with a picosecond pulsed laser (Picoquant; LDH-D-C-485) as an excitation source. The 485 nm laser line from a single mode fiber output of Picoquant laser combining unit first passed the Glan-laser polarizer (Thorlabs, GL5-A) followed by Berek compensator (Newport, Model 5540) to generate circularly polarized light and expanded by a beam expander to fill the back aperture of the objective lens. The samples were excited through a high numerical aperture (NA) oil immersion objective (Olympus, UPlanSApo, ×100, NA 1.40). Raster scanning of the samples was achieved by moving the objective with a precision piezoelectric translation stage (Physik Instrumente, P-733.2CL) controlled by a digital piezo controller (Physik Instrumente, E-710.4CL). Fluorescence from the samples was collected by the same objective, passed through a single-edge laser dichroic beamsplitter (Semrock, FF500 Di01), and was separated from the remaining excitation light by a band-pass filter (Semrock, FF01-609/181). For the measurements of single-particle fluorescence images, fluorescence intensity time trajectories, and fluorescence decay curves, the fluorescence signal from each nanoparticle was detected using a single photon avalanche photodiode (SPAD, PicoQuant, τ-SPAD-50) and recorded by a time-correlated single-photon counting (TCSPC) module (PicoQuant, HydraHarp 400) in the time-tagged time-resolved mode. The brightness of the fluorescence of single particles was estimated by measuring fluorescence images of the individual particles under identical excitation and detection conditions and calculating the integrated intensity of each fluorescence spot. An electron-multiplying charge-

coupled device (CCD) camera (Andor Technology, Newton 970P-BV) equipped with a spectrograph (Andor Technology, SR-303i-B) was used for the measurement of single particle fluorescence spectra under one- or two-photon excitation.

Two-photon excitation single-particle fluorescence microscopy. Two-photon excitation single-particle fluorescence measurements were conducted on a home-built scanning confocal microscope based on an Olympus IX 71 platform[2] equipped with the Ti:Sapphire tunable femtosecond-pulsed laser (Coherent; Chameleon Ultra) operating at 880 nm as a two-photon excitation source. The linearly polarized 880 nm light from the Ti:Sapphire laser was expanded by a beam expander (Thorlabs, GBE05-B), passed through the broadband variable beam intensity attenuator composed of an achromatic half-wave plate (Thorlabs; AHWP10M-980) and Glan-laser polarizer (Thorlabs, GL10-B). Attenuation of the intensity of transmitted beam was accomplished by rotating the half-wave plate. The laser beam with reduced intensity passed through a 780 nm bandpass clean-up filter (Thorlabs, FL780-10) and quarter-wave plate (Thorlabs, AQWP10M-980) to create a circularly polarized light. The intensity of the excitation laser was fine-tuned by neutral density filters (Thorlabs, FW2AND) before introducing into the microscope. The samples were excited in a manner similar to the one-photon excited single-particle fluorescence microscopy experiments. The fluorescence from the samples was collected and detected also in a way similar to the one-photon excited single-particle fluorescence microscopy experiments except that the fluorescence passed through a single-edge laser dichroic beamsplitter (Semrock, FF750-SDi02).

Determination of molar extinction coefficients of the nanoparticles. Molar extinction coefficients (ε) of the nanoparticles were calculated based on their peak absorbance (A) of the ensemble absorption spectra and their molar concentrations estimated by fluorescence correlation spectroscopy (FCS) measurements.[2] The FCS experiment was performed on the custom-built

microscope setup used for single-particle fluorescence microscopy experiments. A 485 nm circularly polarized excitation light (Picoquant; LDH-D-C-485) from the pulsed laser was focused by a water immersion objective (Olympus, UPlanSApo ×60, NA 1.2) onto the sample held in a flow cell. The flow cell was assembled using two clean glass coverslips sealed together with melted parafilm stripes that formed a thin gap between them. All measured nanoparticle samples were additionally filtrated trough 0.02 μm syringe filter (Whatman, Anatop 10) before the experiment. TetraSpec microspheres and fluorescein dye with known concentration (C) and diffusion constant (D) were used to determine the confocal volume (V_{eff}) described by the short (r_0) and long (z_0) axes of an ellipsoid ($V_{\mathrm{eff}} = 4\pi/3 \cdot r_0^2 \cdot z_0$) and validate the performance of the setup (Figure S10). All raw FCS data were analyzed with the equation for particles diffusing in a three-dimensional volume element:

$$G(\tau) = X_{\mathrm{t}}(\tau) \cdot \frac{1}{V_{\mathrm{eff}}} \cdot \frac{1}{(1+\frac{\tau}{\tau_{\mathrm{D}}})} \frac{1}{\sqrt{1+\left(\frac{r_0}{z_0}\right)^2 \cdot (\frac{\tau}{\tau_{\mathrm{D}}})}} \quad (3)$$

$$\tau_{\mathrm{D}} = \frac{\tau_0^2}{4D} \quad (4)$$

$$N = V_{\mathrm{eff}} \cdot C \quad (5)$$

where τ denotes the lag time, τ_{D} is diffusion time of particles in the detection volume, and N is the average particle number in the confocal volume, respectively. $X_{\mathrm{t}}(\tau)$ is the fraction of fluorophores in the triplet state and is described by:

$$X_{\mathrm{t}}(\tau) = 1 - T + T \cdot \exp(-\frac{\tau}{\tau_{\mathrm{triplet}}}) \quad (6)$$

where T and τ_{triplet} are respectively the fraction of the triplet state and the triplet lifetime. The concentration of the nanoparticles (C) was determined from the amplitude of the autocorrelation curve (i.e., $1/(V_{\mathrm{eff}} \cdot C)$) at $\tau = 0$ by fitting the data with equation (3). C was determined for the

solution of the nanoparticles with different dilutions, and the ε value was calculated from the slope of A vs. C plots using the Beer–Lambert law.

Supporting Notes

1. Multi-exponential decaying of the Jdots

A fluorescence decay of the Jdots displayed a deviation from a single-exponential decaying behavior (Figure 1c). The deviation from a single-exponential decay can be interpreted by the size distribution of J-aggregates in each particle because the excited-state lifetime of J-aggregates is determined by the delocalization length of the excited state over the monomers. A similar behavior (i.e., deviation from single exponential decay) has often been observed experimentally.[5] First, although our Jdots have relatively narrow size distribution, each particle has different size. Therefore, the size of the J-aggregates in each particle (and therefore the delocalization length) should be different. Second, as shown in Figure S4, our Jdots sometime consist of multiple crystalline domains. This also causes different size of J-aggregates in each particle. We would like to emphasize though that the deviation from single-exponential decay does not pose any practical issue in practical applications of our Jdots.

2. Exciton superradiance

Superradiance is a collective phenomenon first described by Dicke for an idealized case of non-interacting correlated atoms that were considered as a single quantum system.[6] The basic concept applies to a general N-body system where constituent oscillating dipoles couple together through interaction with shared electromagnetic fields to form a large effective dipole. The presence of

correlated states implies a macroscopic optical polarization proportional to the number (N_c) of atoms or molecules constituting the coherent domain. This leads to an enhancement of the optical transition oscillator strength of the whole system and thus accelerating the radiative decay time (τ_{RAD}) proportionally to the inverse of the number N_c of coherently coupled emitters, i.e., $\tau_{RAD} \propto 1/N_c$. Such enhanced superradiant decay has been demonstrated in a wide variety of systems such as luminescent nanodiamonds,[7] molecular crystals,[8] π-conjugated polymers,[9] quantum dots,[10] and molecular aggregates including H-aggregates and J-aggregates.[11, 12].

3. Hypsochromic shift of the fluorescence of the CzBTCz Jdots compared with that of the CzBTCz monomer in THF

We observed the substantial narrowing of both absorption and fluorescence spectra of CzBTCz upon the nanoparticle formation. Together with the red shifted absorption spectrum and enhanced radiative decay rate, these results strongly suggest the formation of J-aggregates in the CzBTCz nanoparticles (Jdots). Fluorescence spectrum of CzBTCz, on the other hand, showed a blue shift (i.e., hypsochromic shift) upon the particle formation. This is uncommon for J-aggregates[11, 13] since an excited-state energy of J-aggregates is normally lower than that of the corresponding monomer. An intramolecular charge transfer (ICT) between the carbazole (Cz) donor (D) and benzothiadiazole (BT) acceptor (A) moieties is responsible for the absorption of CzBTCz in the visible wavelength region.[1] The ICT state is also responsible for the fluorescence of CzBTCz.[1] Many ICT molecules exhibit a twist between donor and acceptor moieties in their excited state (i.e., twisted intramolecular charge transfer (TICT) state). In general, a TICT state has lower energy compared with an excited ICT state initially formed upon photoexcitation in a polar environment. Therefore, fluorescence from the excited state of D-A-type molecules often displays

a red shift in a polar environment that stabilizes the TICT state significantly. Indeed, fluorescence spectra of CzBTCz displayed red shifts with increase in the polarity of the medium, which is indicative of increased solvation of the TICT states.[1] However fluorescence spectra of CzBTCz in the Jdots resemble the spectra of CzBTCz in cyclohexane (nonpolar solvent suppressing TICT emission, see Figure S1), indicating negligible effect of water polarity on the fluorescence properties of the Jdots (i.e. formation of the TICT state is suppressed). In addition, the fluorescence spectrum of the Jdots is significantly narrower compared with the spectrum of the CzBTCz monomer in nonpolar solvent. This behavior is explained by highly-ordered packing of CzBTCz in the Jdots (as expected for J-aggregates), leading to fluorescence spectral narrowing. Together, the spectral blue shift and narrowing upon the Jdots formation strongly suggest that the formation of the TICT state in the Jdots is suppressed and this is not affected by the high polarity water environment. A similar blue shift of fluorescence was observed for the CzBT nanoparticles that do not form J-aggregates upon the particle formation (Figure S6b), also supporting the model. We note that the alkyl chains in the CzBTCz monomer are not an obstacle as previous study indicated that the presence of long alkyl substituent supports the formation of J-aggregates.[14, 15]

4. Estimation of the peak molar extinction coefficient (*ε*) of the CzBTCz Jdots using fluorescence correlation spectroscopy (FCS)

The peak molar extinction coefficient (*ε*) of the nanoparticles (NPs) was calculated by estimating the concentration of the nanoparticles in the solution using FCS. A precise determination of the concentration of the NPs in solution is nontrivial. While FCS is most reliable and direct method for the determination of the NPs' concentration, the estimated concentration could be biased by a presence of a small number of very large particles (or aggregates of the particles) because the contribution of each NP to the autocorrelation curve scales with the square of the brightness. That

means a small number of very large particles could dominate the autocorrelation function. Therefore, a threshold was applied to the intensity time trajectory to remove a small number of very large bursts, which minimizes the influence of very large particles on the estimation of the concentration of the NPs in the solution (Figure SN1).

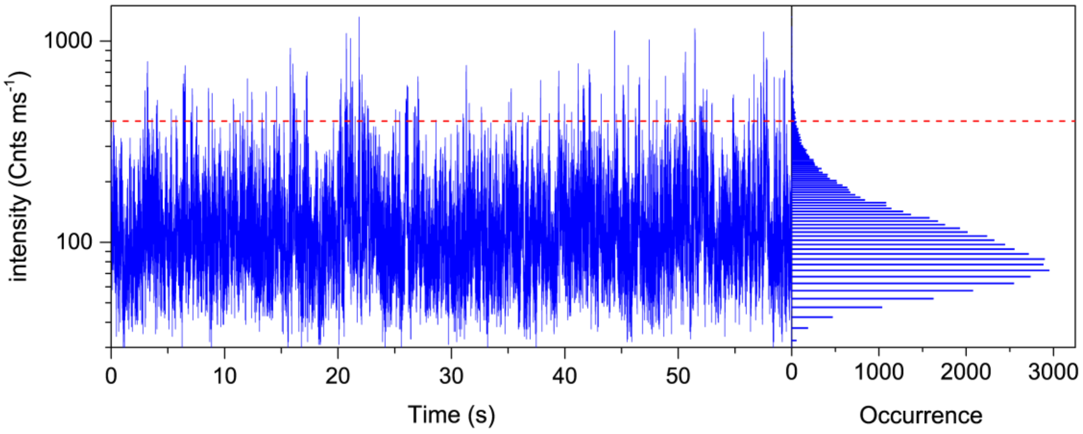

Figure SN1. Fluorescence correlation spectroscopy (FCS) analysis of the CzBTCz Jdots in water. (left) A typical fluorescence intensity time trajectory obtained from a diluted aqueous solution of the CzBTCz Jdots. An autocorrelation curve was calculated using the data points below a threshold indicated by red dashed line. The threshold was set at 3σ (σ: standard deviation) of the intensity distribution. (right) Frequency histogram of the intensity distribution obtained from the intensity time trajectory.

5. Theoretical description of enhanced peak molar extinction coefficient (ε) per monomer in J-aggregates

An enhancement of the peak molar extinction coefficient (ε) or two-photon absorption cross section (δ) in J-aggregates compared with that of the monomer has been reported for many molecules.[16, 17] According to photophysics theory, an oscillator strength (f), which corresponds to the probability of light absorption by a molecule, is proportional to the integrated molar extinction

coefficient (ε_{int}).[18] Since f is proportional to the square of the transition dipole moment (μ), ε_{int} is proportional to μ^2. When N number of molecules form a J-aggregate, the total transition dipole moment of the aggregate (μ_{agg}) is described by $\mu_{agg} = \sqrt{N}\mu$.[19] Therefore, ε_{int} of the J-aggregate (ε_{int}^J) is described by $\varepsilon_{int}^J \propto N\mu^2$. Since the total ε_{int} of the N number of the monomer molecules is also described by $N\varepsilon_{int} \propto N\mu^2$, there is no net enhancement of ε_{int} per each molecule in the J-aggregate.[20] The peak molar extinction coefficient (ε) is, thus, inversely proportional to the spectral width of the absorption spectra. The narrower the absorption spectrum is, the larger the ε value is. This means that an effective enhancement of ε per each molecule is predicted when J-aggregates are formed. The experimentally observed spectral widths of the monomer CzBTCz (3981 cm^{-1}) and CzBTCz Jdots (1114 cm^{-1}) suggest 3.6 fold enhancement in the ε value upon the Jdots formation.

6. Calculation of the exciton delocalization length (N_{del}, number of molecules in an aggregate over which electron is delocalized)

An exciton delocalization length (N_{del}) in J-aggregates is experimentally determined by equation SN1,[21]

$$N_{del} \approx \frac{3}{2}\left(\frac{\Delta\nu_{0.5}{}^{m}}{\Delta\nu_{0.5}{}^{J}}\right)^2 \quad \text{(SN1)}$$

where $\Delta\nu_{0.5}{}^m$ and $\Delta\nu_{0.5}{}^J$ denote the half-width values of the monomer and J-aggregate absorption bands. According to the absorption spectra of CzBTCz in THF and the CzBTCz Jdots (Fig. 1b), N_{del} in the CzBTCz Jdots is estimated to be about 19.

Alternatively, N_{del} in J-aggregates is experimentally determined by equation SN2 or SN3,[21, 22]

$$N_{del} \approx \frac{\pi^2}{8}\frac{\tau_r{}^{m}}{\tau_r{}^{J}} \quad \text{(SN2)}$$

$$N_{\mathrm{del}} \approx \frac{k_{\mathrm{r}}{}^{\mathrm{J}}}{k_{\mathrm{r}}{}^{\mathrm{m}}}\left(\frac{\lambda_{\mathrm{J}}}{\lambda_{\mathrm{m}}}\right) \qquad \text{(SN3)}$$

where τ_r^m and τ_r^J denote the radiative lifetimes ($1/k_r^m$ and $1/k_r^J$) of the monomer and J-aggregate. λ_m and λ_m are the peak absorption wavelength of the monomer and J-aggregate. According to the k_r and λ values in Table 1, N_{del} in the CzBTCz Jdots is estimated to be about 5 and 4.6 for equation SN2 and SN3, respectively.

The N_{del} values estimated by SN1 and SN2 (or SN3) correspond to the number of the molecules where the electron is delocalized along the aggregate in the ground and excited state, respectively. The discrepancy between N_{del} values estimated by absorption and fluorescence have been reported previously in many papers.[23-28] Since the enhancement of the molar extinction coefficient upon the particle formation is most important point in our discussion, and since the absorption spectra are most reliable data, N_{del} = 19 is the most relevant value for our discussion.

7. Factors affecting the calculation of the peak molar extinction coefficient (ε) of the CzBTCz Jdots

In addition to the spectral narrowing-induced enhancement of the peak molar extinction coefficient (ε) described in Supporting Note 4, a twist angle between the carbazole (Cz) and benzothiadiazole (BT) moieties should also contribute to the observed enhancement in the ε value. Our previous study suggested that the oscillator strength (and therefore ε value) of the intramolecular charge transfer (ICT) absorption of CzBTCz depends on the twist angle between the Cz and BT moieties (i.e., the smaller the twist angle is, the larger the ε value is).[1] In addition, polymer dots fabricated from the $(CzBT)_n$ conjugated polymer molecule in a way similar to the CzBTCz Jdots showed a smaller twist angle between the Cz and BT moieties upon the particle

formation.[2] Therefore, it is likely that the CzBTCz molecule has a smaller twist angle between the Cz and BT moieties upon the Jdots formation, which results in a larger ε value.

8. Comparison of the fluorescence quantum yield of the CzBTCz Jdots and J-aggregates of cyanine dyes

Cyanine dyes (e.g. pseudoisocyanine (PIC) dye) show very low ϕ_{fl} in water (e.g. ϕ_{fl} = 0.025 in water for PIC).[29, 30] Only when the dyes are incorporated into matrices (e.g. films, surfactants), they show relatively large ϕ_{fl} (e.g. ϕ_{fl} = 0.38 for PIC mixed with cetylpyridinium bromide (CPB) surfactant at the molar ratio of 1:4). These results strongly suggest that the pure J-aggregates of cyanine dyes formed in water show very low ϕ_{fl}, which is in stark contrast to near-unity ϕ_{fl} (ϕ_{fl} = 0.95) of our Jdots.[29]

9. Number of CzBTCz monomer molecules in each Jdot

We estimated the number of monomers in each Jdots (N) based on the volume of the Jdots (V = 22.5 nm^3), molecular weight of the monomer (MW = 1500 g mol^{-1}), and density of the Jdots (density of carbon materials is in the range of Ro = 1.8-3.5 g cm^{-3}). Since the number of monomers in each Jdot is described by $N = (V \times \text{Ro}) / \text{MW}$, N is estimated to be in the range of $N = 16 – 32$. N=19 is obtained when Ro = 2.1 g cm^{-3}. Since our particles consist of J-aggregates, not random aggregates, the density could be higher than other conventional amorphous carbon-based nanoparticles. Together, our calculation strongly suggests that most of our Jdots consist of at least 19 monomers.

The number of CzBTCz monomer molecules in each Jdots could, in principle, be estimated by X-ray data; X-ray crystallographic data or XRD data. However, these characterizations require

either a large crystal or a large amount of tightly packed Jdots. The first option is excluded since macroscopic crystals are irrelevant to this study, which aimed to develop sub-5 nm size particles. The second option does not work well because the spectroscopic properties of the Jdots change when they are packed tightly in the solid state (Figure SN2). This is because either 1) the molecular packing in the particles changes during the formation of the tightly packed Jdots in the solid state (through interparticle interactions) or 2) interparticle interactions change electronic delocalization over the J-aggregates.

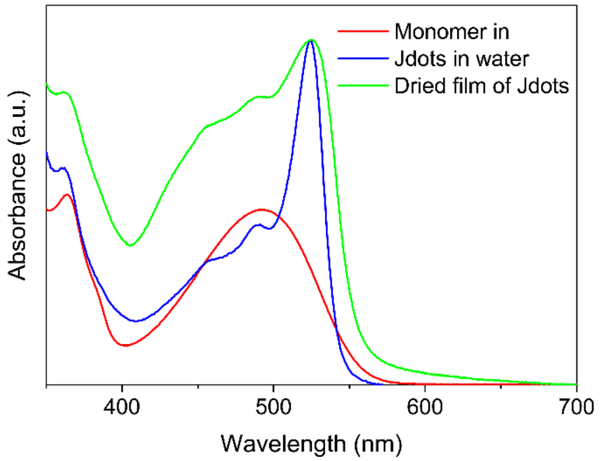

Figure SN2. Steady state absorption spectra of CzBTCz in THF (red), CzBTCz Jdots in water (blue), and CzBTCz Jdots in a dried film.

10. Possibility of the generation of carbon nanoparticles in the HRTEM measurement

We conducted selected-area electron diffraction (SAED) measurement in order to discuss the periodical structures (i.e., polycrystalline structures) of the Jdots. The SAED pattern obtained from the Jdots (Figure 2a inset) exhibits sharp ring patterns (i.e., polycrystalline structures). In contradiction, the SAED pattern of the CzBT nanoparticles (Figure 2b inset) confirmed their amorphous character (as expected from spectroscopy data). Both samples were exposed to same

electron beam intensity during the measurements. These results exclude the effect of the electron beam exposure on the observed polycrystalline structures of the CzBTCz Jdots.

XRD is an alternative method to characterize the periodical structures. However, SAED measurement can be performed easily with limited electron-beam exposure, providing information about internal structures of samples in a similar way as powder XRD does (lattice spacing) from an ensemble of particles but on much smaller scale of samples. XRD and SAED both are governed by the Bragg equation and basically differs only in wavelength of diffraction beam.

SAED usually probes a small area (in sub µm diameter range). In addition, electrons interact much stronger with small matters than X-ray does, providing substantially higher diffracted intensities. Thus, the SAED patterns can be obtained using smaller amount/less density of samples compared with XRD measurement. Indeed, we were able to obtain the TEM/SAED data of our Jdots using relatively small amount of the Jdots in diluted condition (i.e., a drop of the Jdots suspension in water (sub µM concentration) was casted on TEM grids). As is evident from Figure 2a, interactions between the Jdots were negligible at this condition.

On the other hand, the XRD measurement requires a greater amount of the material, and thus long-term concentration of the nanoparticles suspension with further drying step is necessary. This treatment of the sample causes changes in their structures (due to the interactions between the nanoparticles). Thus, the XRD measurement won't provide accurate structure of our Jdots.

In addition, X-ray diffraction experiments for nanocrystals often suffer from significant diminishing of peak intensities due to the peak broadening and overlapping. For instance, many crystalline materials are too small in grain size and are too imperfect in periodicity for an X-ray analysis to be performed, but are applicable for electron-microscopic observations. TEM is the

technique that simultaneously provides a microscope image and a diffraction pattern at atomic resolution with relatively small amount of samples, unlike XRD that requires a large amount of samples.

With regard to the 0.28 nm lattice fringe observed in the HRTEM measurement, we cannot rule out the possibility that graphene-like carbon nanoparticles are generated by the electron beam irradiation during the measurement. However, we would like to emphasize that similar morphologic structures, including J-aggregates, were observed under electron microscope for indocyanine green in water,[31] pseudoisocyanine in water,[32] and corannulene-cyclodextrin nanoassemblies,[33] indicating that the lattice fringe observed in our experiment reflects the polycrystalline structures of the Jdots.

11. Characterization of the fluorescence brightness of the Jdots

The fluorescence brightness of the CzBTCz Jdots was further characterized by comparing its intrinsic brightness, $\varepsilon\phi_{\mathrm{fl}}$, with other frequently used fluorophores (Figure SN3). The data clearly shows that the intrinsic brightness of the Jdots is approximately two orders of magnitude larger than that of organic fluorophores (rhodamine 6G (R6G) and *N,N'*-Bis(2,6-dimethylphenyl)perylene-3,4,9,10-tetracarboxylic diimide (DXP)) as well as Qdots545 (QD545) that emit photoluminescence in the similar wavelength region.

The fluorescence brightness of the CzBTCz Jdots was also experimentally characterized by comparing its experimentally obtained fluorescence intensity with other frequently used fluorophores and fluorescent nanoparticles (Figure SN4). The data demonstrated that the Jdots show more than two orders of magnitude brighter fluorescence compared with the organic dye (R6G) and show fifty times brighter fluorescence compared with Qdots545 (QD545) that emit

photoluminescence in the similar wavelength region. The result further demonstrated the 3.5-nm Jdots exhibit fluorescence brightness comparable to 100-nm dye doped polymer nanoparticles (TetraSpec fluorescent microspheres,TS).

We also evaluated the fluorescence brightness of the CzBTCz Jdots using a brightness parameter (*B*) that is defined by the experimentally detected photon numbers per second normalized by the excitation power density, which is not affected by experimental conditions (Figure SN5). The data shows that the *B* value obtained for the CzBTCz Jdots is more than two orders of magnitude larger than those obtained for the organic dyes (R6G and DPX) and fifty times larger than that obtained for Qdots545 (QD545) that emit photoluminescence in the similar wavelength region. All these data demonstrate that the CzBTCz Jdots exhibit much brighter fluorescence compared with conventional organic dyes and photoluminescent nanoparticles.

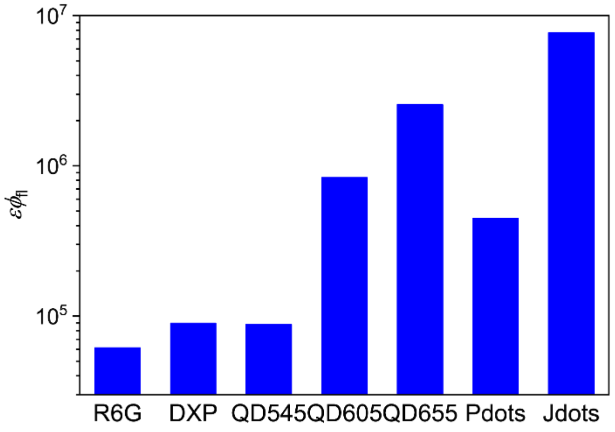

Figure SN3. Intrinsic brightness of frequently used fluorophores. Intrinsic brightness (product of molar extinction coefficient and fluorescence quantum yield) of rhodamine 6G (R6G), N,N′-Bis(2,6-dimethylphenyl)perylene-3,4,9,10-tetracarboxylic diimide (DXP), Qdots545 (QD545), Qdots605 (QD605), Qdots655 (QD655), (CzBT)n polymer dots (Pdots), and CzBTCz Jdots.

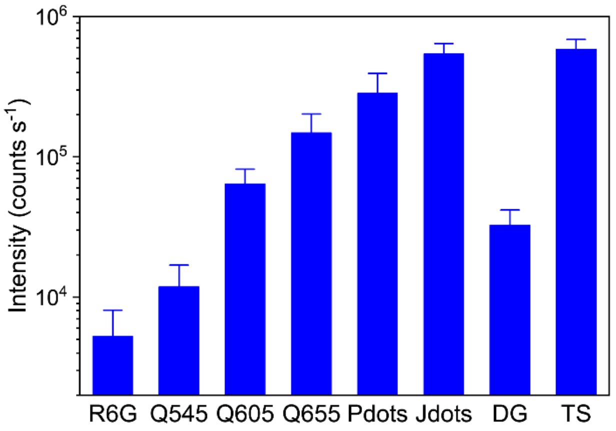

Figure SN4. Experimentally obtained fluorescence brightness of frequently used fluorophores and fluorescent nanoparticles. Mean fluorescence intensities of individual rhodamine 6G (R6G), Qdots545 (QD545), Qdots605 (QD605), Qdots655 (QD655), $(CzBT)_n$ polymer dots (Pdots), CzBTCz Jdots (Jdots), 50-nm dye doped polymer nanoparticles (DG), and 100-nm dye doped polymer nanoparticles (TetraSpec fluorescent microspheres, TS) obtained under identical experimental conditions (485 nm excitation, 424 W cm^{-2} excitation power). Error bars show standard deviations determined by 2768, 1275, 586, 1663, 611, 1871, 1639 and 401 molecules/particles for the R6G, QD545, QD605, QD655, Pdots, Jdots, DG, and TS, respectively.

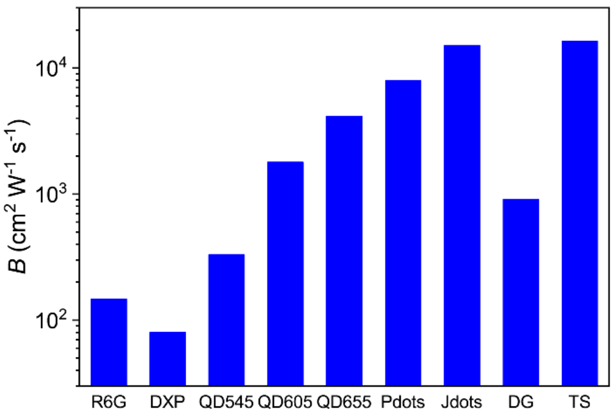

Figure SN5. Experimentally obtained brightness parameter B of frequently used fluorophores and fluorescent nanoparticles. B values of rhodamine 6G (R6G), N,N′-Bis(2,6-dimethylphenyl)perylene-3,4,9,10-tetracarboxylic diimide (DXP), Qdots545 (QD545), Qdots605 (QD605), Qdots655 (QD655), (CzBT)n polymer dots (Pdots), CzBTCz Jdots (Jdots), 50-nm dye doped polymer nanoparticles (DG), and 100-nm dye doped polymer nanoparticles (TS). Note that the photon numbers were calculated using the obtained count rates and overall collection efficiency of the microscope setup that is estimated to be 7.9 % (50 % transmission of the objective lens, 60 % transmission of other optics, 44 % transmission through the pinhole, and 60 % quantum efficiency of the photodetector). The B value of DXP is quoted from reference 34.[34]

Supporting Figures

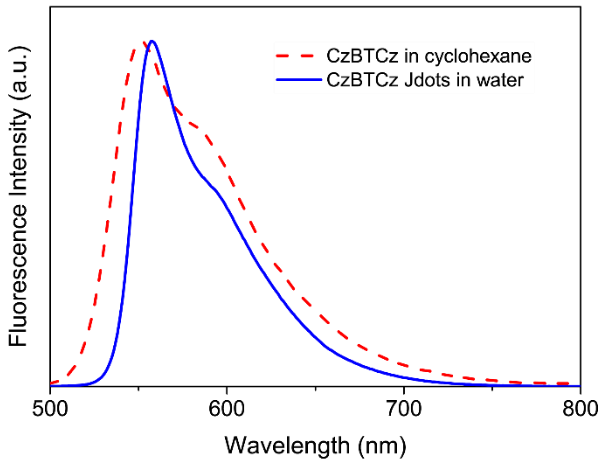

Figure S1. Comparison of fluorescence spectra of CzBTCz. Ensemble fluorescence spectra of CzBTCz Jdots dispersed in water (blue) and CzBTCz in cyclohexane (red dashed line). The spectra were recorded upon 450 nm excitation.

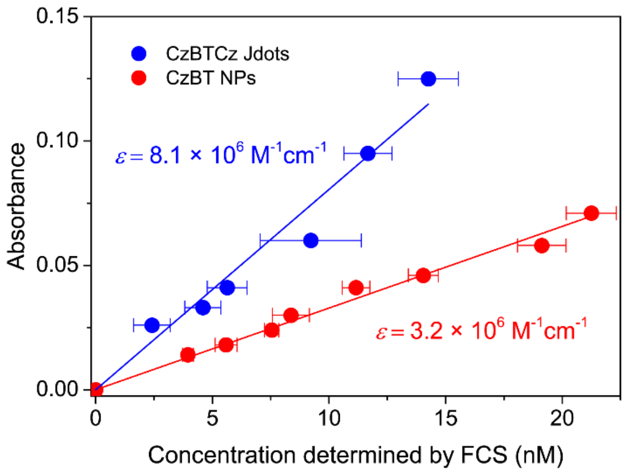

Figure S2. Determination of molar extinction coefficients (ε) of the nanoparticles using fluorescence correlation spectroscopy (FCS). Absorbance vs. concentration (C) plots of the CzBTCz Jdots and CzBT NPs. The C values were determined by the FCS measurements. The error bars show standard deviations determined by ten repeated measurements. Solid lines show linear fit of the data. The ε values were calculated from the slopes of the linear fit.

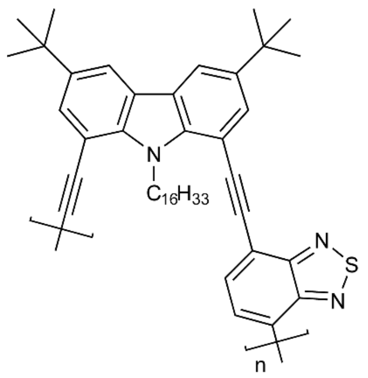

Figure S3. Chemical structure of the $(CzBT)_n$ conjugated polymer.[2]

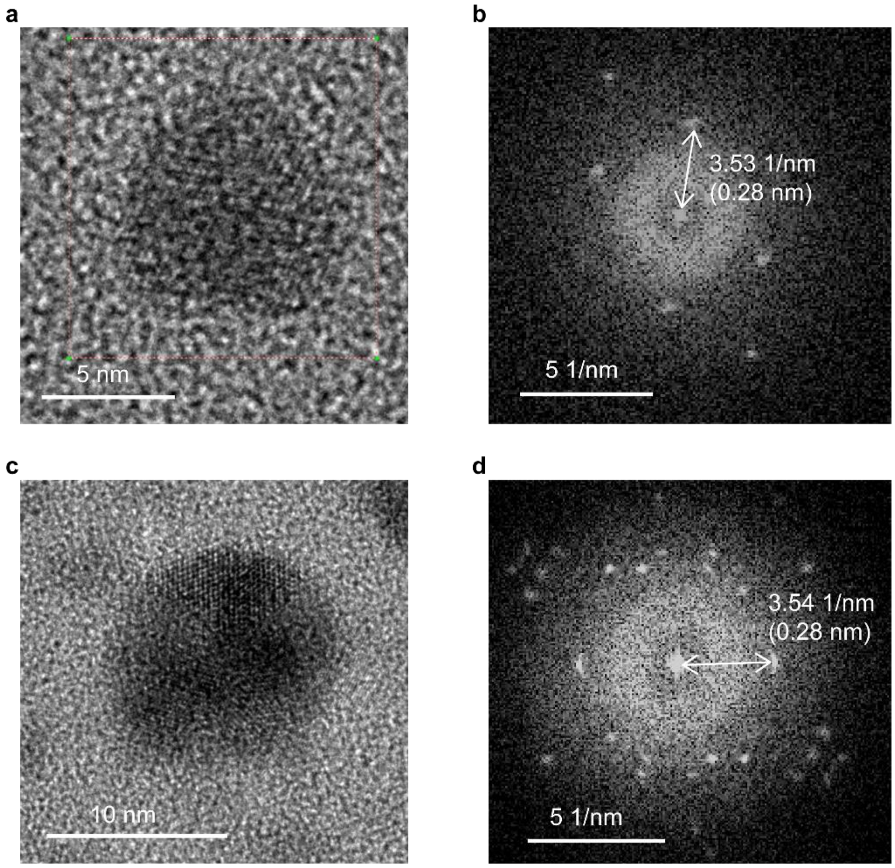

Figure S4. TEM images of the CzBTCz Jdots. TEM image of the CzBTCz Jdots that consist of (a) three crystalline domains and (c) large number of crystalline domains. Fast Fourier transform (FFT) images calculated from (b) the TEM images displayed in (a) and (d) the TEM images displayed in (c).

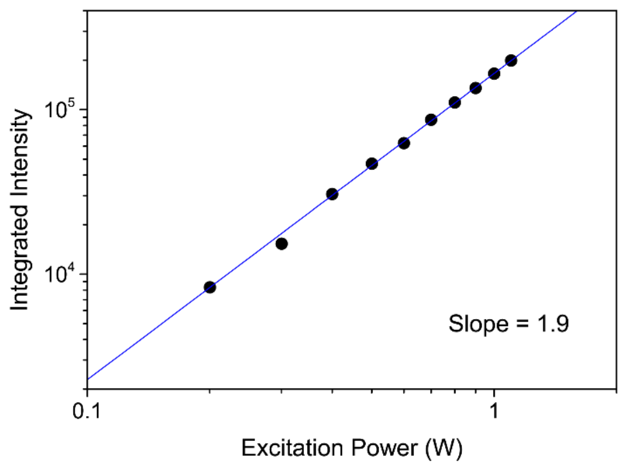

Figure S5. Excitation power dependent fluorescence intensity of the CzBTCz Jdots. The CzBTCz Jdots in water were excited at 880 nm with a femtosecond pulsed Ti: Sapphire laser. Integrated intensity of the fluorescence spectrum obtained at each excitation power was plotted against the excitation power. The blue line shows a linear fit of the log-log plot of the data. The slope obtained from the fitting (slope = 1.9) is close to the theoretically predicted slope for two-photon absorption (slope = 2).

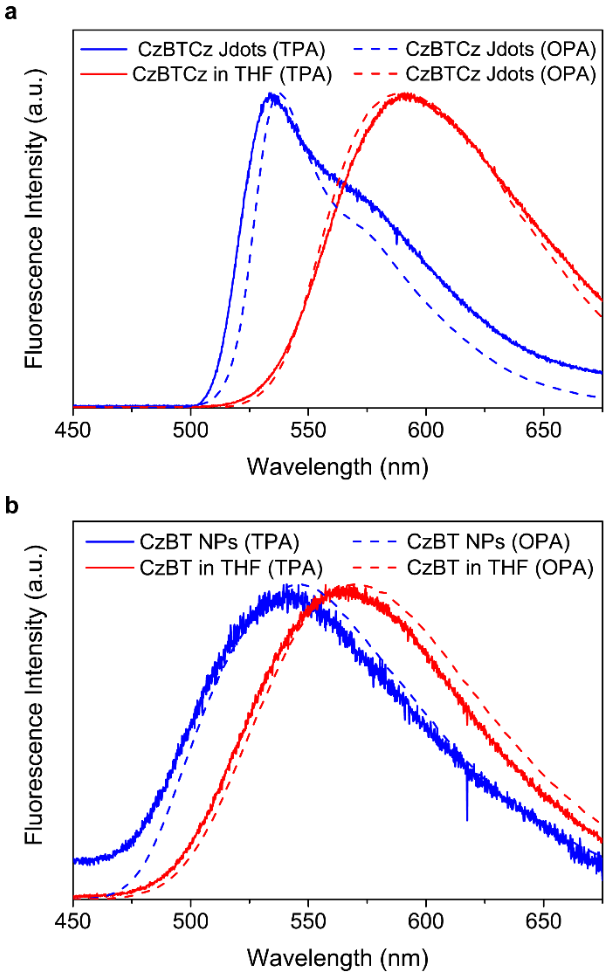

Figure S6. Ensemble two-photon absorption (TPA)-induced fluorescence spectra of the nanoparticles. (a) Fluorescence spectra of CzBTCz Jdots in water (blue) and CzBTCz in THF (red). One-photon absorption (OPA)-induced fluorescence of CzBTCz Jdots in water (dashed blue line) and CzBTCz in THF (dashed red line) are displayed for reference. (b) Fluorescence spectra of CzBT nanoparticles (NPs) in water (blue) and CzBT in THF (red). One-photon absorption (OPA)-induced fluorescence of CzBT NPs in water (dashed blue line) and CzBT in THF (dashed red line) are displayed for reference. The TPA-induced fluorescence spectra were recorded upon excitation at 880 nm with a femtosecond pulsed Ti: Sapphire laser. The OPA-induced fluorescence spectra were recorded upon excitation at 450 nm.

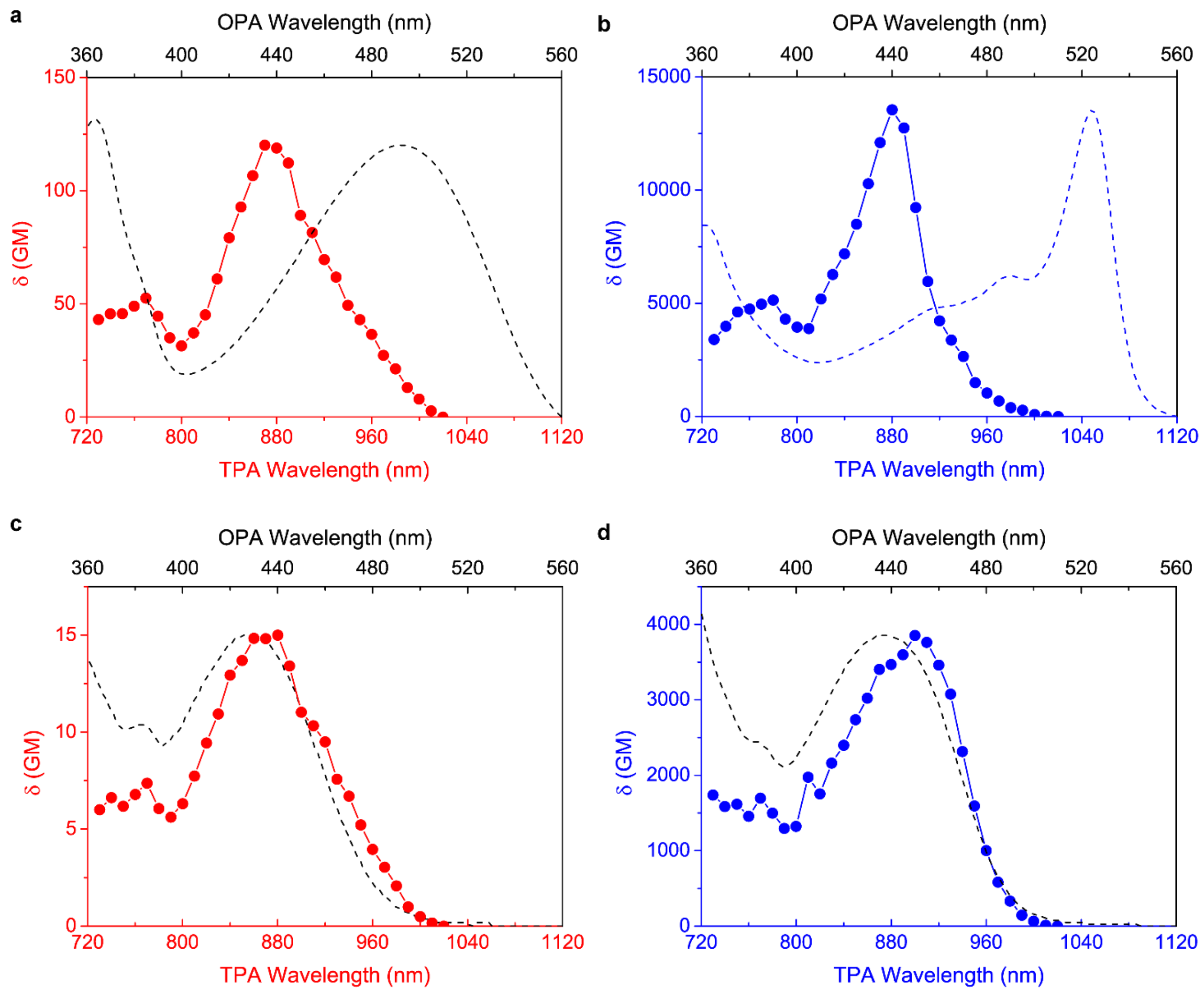

Figure S7. Two-photon absorption (TPA) properties of the CzBTCz Jdots. (a) TPA spectrum of CzBTCz in THF. Dashed line shows a one-photon absorption (OPA) spectrum of CzBTCz in THF. (b) TPA spectrum of the CzBTCz nanoparticles dispersed in water. Dashed line shows the OPA spectrum of the CzBTCz nanoparticles dispersed in water. (c) TPA spectrum of CzBT in THF. Dashed line shows the OPA spectrum of CzBT in THF. (d) TPA spectrum of the CzBT nanoparticles dispersed in water. Dashed line shows the OPA spectrum of the CzBT nanoparticles dispersed in water. Note that the same figure is shown in Figure 3 in a logarithmic scale. The spectra displayed in a linear scale in this figure are for visual inspection of spectral widths and shifts.

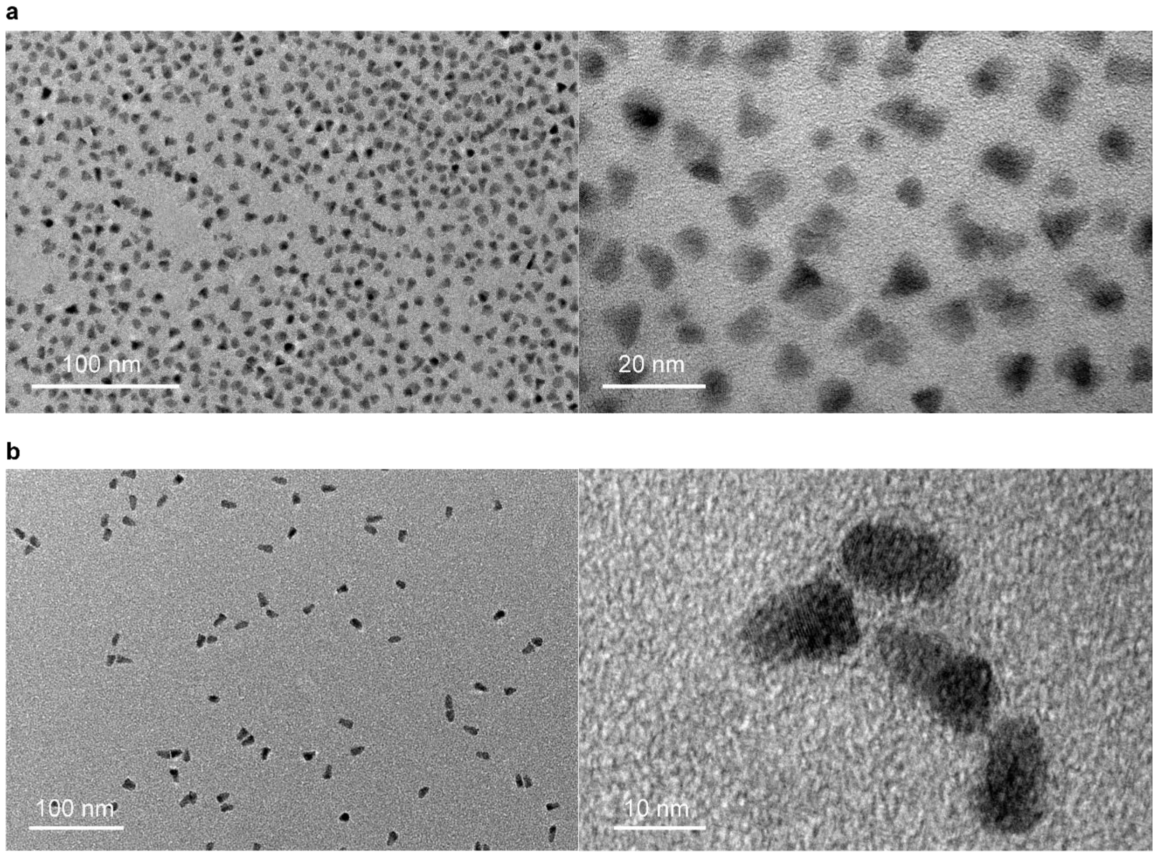

Figure S8. TEM images of semiconductor quantum dots. TEM images of (a) Qdots 605 (QD605) and (b) Qdots 655 (QD655) quantum dots. The diameters of the particles along long and short axes were estimated to be 8 and 5 nm for QD605 and 12 and 6 nm for QD655.

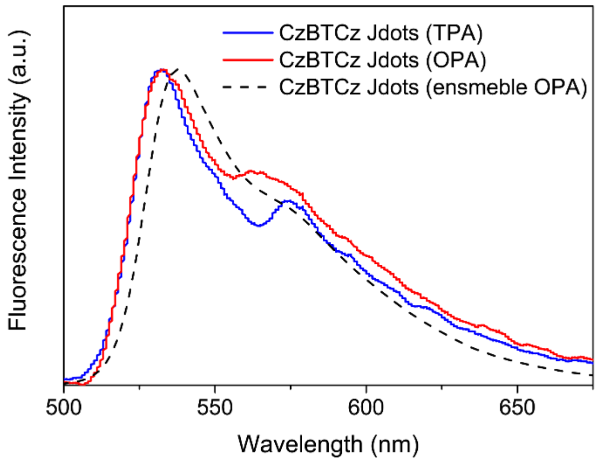

Figure S9. Single-particle two-photon absorption (TPA)-induced fluorescence spectra of CzBTCz Jdots. Typical example of TPA-induced fluorescence spectrum obtained from a single CzBTCz Jdot (blue line). Single-particle (red line) and ensemble (dashed line) fluorescence spectra of the CzBTCz Jdots obtained by OPA-induced fluorescence are displayed for reference.

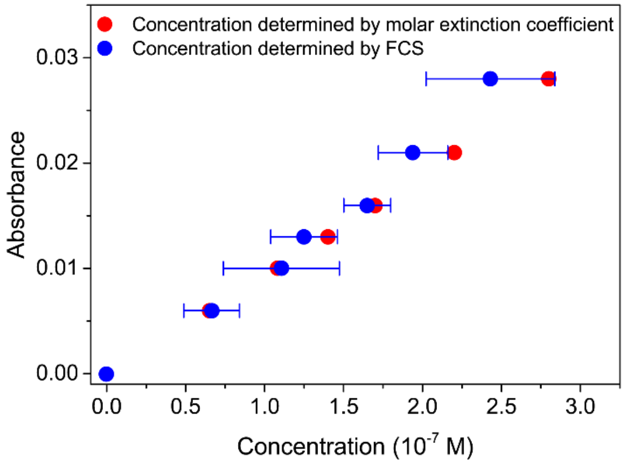

Figure S10. Validation of the fluorescence correlation spectroscopy (FCS) measurement. Absorbance of a reference sample, fluorescein, at known concentrations (red) and absorbance of fluorescein samples whose concentrations were estimated by the FCS experiment (blue). The error bars show standard deviations determined by five repeated measurements.

Supporting Table

Table S1. Comparison of the fluorescence brightness of the CzBTCz Jdots with other fluorescent nanoparticles.

	Size (nm)[a]	Volume (nm^3)	ε (M^{-1} cm^{-1})	ϕ_{fl}	δ (GM)	$\varepsilon\phi_{fl}/V$	$\delta\phi_{fl}/V$
QD545[b]	3.8	29.7[e]	2.1×10^5	0.42	6.00×10^3	2970	85.0
QD605[b]	5.0, 8.0[d]	105[f]	1.1×10^6	0.76	2.62×10^4	7980	190
QD655[b]	6.0, 12[d]	226[f]	2.9×10^6	0.88	7.02×10^4	11300	273
$(CzBT)_n$ Pdots[b,c]	3.0	14.1[e]	2.7×10^6	0.16	3.96×10^4	31700	448
CzBTCz Jdots[b]	3.5	22.5[e]	8.1×10^6	0.95	1.35×10^4	343000	573

a: diameter of the particles determined by TEM, *b*: measured in water, *c*: polymer dots (Pdots), *d*: diameters of the short and long axes of the ellipsoid, *e*: volume of the sphere with a diameter shown in the Size column, *f*: volume of the ellipsoid with diameters along the short and long axes shown in the Size column.

Supporting References

1. Habuchi, S.; Fujita, H.; Michinobu, T.; Vacha, M., Twist Angle Plays an Important Role in Photophysical Properties of a Donor-Acceptor-Type Conjugated Polymer: A Combined Ensemble and Single-Molecule Study. *J. Phys. Chem. B* **2011,** *115* (49), 14404-14415.

2. Piwonski, H.; Michinobu, T.; Habuchi, S., Controlling Photophysical Properties of Ultrasmall Conjugated Polymer Nanoparticles Through Polymer Chain Packing. *Nat. Commun.* **2017,** *8*, 15256.

3. Xu, C.; Webb, W. W., Measurement of two-photon excitation cross sections of molecular fluorophores with data from 690 to 1050 nm. *J. Opt. Soc. Am. B-Opt. Phys.* **1996,** *13* (3), 481-491.

4. He, G. S.; Tan, L. S.; Zheng, Q.; Prasad, P. N., Multiphoton absorbing materials: Molecular designs, characterizations, and applications. *Chem. Rev.* **2008,** *108* (4), 1245-1330.

5. Spano, F. C.; Mukamel, S., Superradiance in molecular aggregates. *J. Chem. Phys.* **1989,** *91* (2), 683-700.

6. Dicke, R. H., Coherence in spontaneous radiation processes. *Phys. Rev.* **1954,** *93* (1), 99-110.

7. Bradac, C.; Johnsson, M. T.; van Breugel, M.; Baragiola, B.; Martin, R.; Juan, M. L.; Brennen, G. K.; Volz, T., Room-temperature spontaneous superradiance from single diamond nanocrystals. *Nat. Commun.* **2017,** *8*, 1205.

8. Knoester, J., Exciton superradiance in molecular-crystal slabs. *J. Lumines.* **1992,** *53* (1-6), 101-104.

9. Khachatryan, B.; Nguyen, T. D.; Vardeny, Z. V.; Ehrenfreund, E., Phosphorescence superradiance in a Pt-containing pi-conjugated polymer. *Phys. Rev. B* **2012,** *86* (19), 195203.

10. Scheibner, M.; Schmidt, T.; Worschech, L.; Forchel, A.; Bacher, G.; Passow, T.; Hommel, D., Superradiance of quantum dots. *Nat. Phys.* **2007,** *3* (2), 106-110.

11. Bricks, J. L.; Slominskii, Y. L.; Panas, I. D.; Demchenko, A. P., Fluorescent J-aggregates of cyanine dyes: basic research and applications review. *Methods Appl. Fluoresc.* **2018,** *6* (1), 012001.

12. Meinardi, F.; Cerminara, M.; Sassella, A.; Bonifacio, R.; Tubino, R., Superradiance in molecular H aggregates. *Phys. Rev. Lett.* **2003,** *91* (24), 247401.

13. Wurthner, F.; Kaiser, T. E.; Saha-Moller, C. R., J-Aggregates: From Serendipitous Discovery to Supramolecular Engineering of Functional Dye Materials. *Angew. Chem. Int. Ed.* **2011,** *50* (15), 3376-3410.

14. De Rossi., U.; Moll, J.; Kriwanek, J.; Daehne, S., Influence of the n-alkyl chain length on the J-aggregation behavior of a cyannine dye. *J. Fluoresc.* **1993,** *4* (1), 53-55.

15. Shapiro, B. I., Molecular assemblies of polymethine dyes. *Russ. Chem. Rev.* **2006,** *75* (5), 433-456.

16. Collini, E.; Ferrante, C.; Bozio, R., Strong enhancement of the two-photon absorption of tetrakis(4-sulfonatophenyl)porphyrin diacid in water upon aggregation. *J. Phys. Chem. B* **2005,** *109* (1), 2-5.

17. Ray, P. C.; Sainudeen, Z., Very large infrared two-photon absorption cross section of asymmetric zinc porphyrin aggregates: Role of intermolecular interaction and donor-acceptor strengths. *J. Phys. Chem. A* **2006,** *110* (44), 12342-12347.

18. Turro, N. J., Radiative Transitions - The Absoprtion and Emission of Light. In *Modern Molecular Photochemistry*, University Science Books: Sausalito, 1991; pp 76-152.

19. McRae, E. G.; Kasha, M., Enhancement of phosphorescence ability upon aggregation of dye molecules. *J. Chem. Phys.* **1958,** *28* (4), 721-722.

20. Devoe, H., Optical properties of molecular aggregates. 1. classical model of electronic absorption and refraction. *J. Chem. Phys.* **1964,** *41* (2), 393-400.

21. Losytskyy, M. Y.; Yashchuk, V. M., Fluorescent J-aggregates and their biological applications. In *Advanced Fluorescence Reporters in Chemistry and Biology II*, Demchenko, A. P., Ed. Springer: Berlin Heidelberg, 2010; pp 135-157.

22. Kamalov, V. F.; Struganova, I. A.; Yoshihara, K., Temperature dependent radiative lifetime of J-aggregates. *J. Phys. Chem.* **1996,** *100* (21), 8640-8644.

23. Malyukin, Y. V.; Sorokin, A. V.; Semynozhenko, V. P., Features of exciton dynamics in molecular nanoclusters (J-aggregates): Exciton self-trapping. *Low Temp. Phys.* **2016,** *42* (6), 429-440.

24. Tani, K.; Matsuzaki, K.; Kodama, Y.; Fukita, M.; Kodaira, T.; Horiuchi, H.; Okutsu, T.; Hiratsuka, H., Photophysical property of the J-aggregate thin film of an oxacyanine dye prepared by the spin-coating method and enhancement of its photostability by use of polydimethylsilane. *J. Photochem. Photobiol. A-Chem.* **2008,** *199* (2-3), 150-155.

25. Obara, Y.; Saitoh, K.; Oda, M.; Tani, T., Room-Temperature Fluorescence Lifetime of Pseudoisocyanine (PIC) J Excitons with Various Aggregate Morphologies in Relation to Microcavity Polariton Formation. *Int. J. Mol. Sci.* **2012,** *13* (5), 5851-5865.

26. Hestand, N. J.; Spano, F. C., Expanded Theory of H- and J-Molecular Aggregates: The Effects of Vibronic Coupling and Intermolecular Charge Transfer. *Chem. Rev.* **2018,** *118* (15), 7069-7163.

27. Sorokin, A. V.; Pereverzev, N. V.; Grankina, II; Yefimova, S. L.; Malyukin, Y. V., Evidence of Exciton Self-Trapping in Pseudoisocyanine J-Aggregates Formed in Layered Polymer Films. *J. Phys. Chem. C* **2015,** *119* (49), 27865-27873.

28. Lutsyk, P.; Piryatinski, Y.; Shandura, M.; AlAraimi, M.; Tesa, M.; Arnaoutakis, G. E.; Melvin, A. A.; Kachkovsky, O.; Verbitsky, A.; Rozhin, A., Self-Assembly for Two Types of J-Aggregates: cis-Isomers of Dye on the Carbon Nanotube Surface and Free Aggregates of Dye trans-Isomers. *J. Phys. Chem. C* **2019,** *123* (32), 19903-19911.

29. Guralchuk, G. Y.; Katrunov, I. K.; Grynyov, R. S.; Sorokin, A. V.; Yefimova, S. L.; Borovoy, I. A.; Malyukin, Y. V., Anomalous surfactant-induced enhancement of luminescence quantum yield of cyanine dye J-aggregates. *J. Phys. Chem. C* **2008,** *112* (38), 14762-14768.

30. Horng, M. L.; Quitevis, E. L., Excited-state dynamics of polymer-bound J-aggregates. *J. Phys. Chem.* **1993,** *97* (47), 12408-12415.

31. Zweck, J.; Penzkofer, A., Microstructure of indocyanine green J-aggregates in aqueous solution. *Chem. Phys.* **2001,** *269* (1-3), 399-409.

32. von Berlepsch, H.; Bottcher, C.; Dahne, L., Structure of J-aggregates of pseudoisocyanine dye in aqueous solution. *J. Phys. Chem. B* **2000,** *104* (37), 8792-8799.

33. Sreejith, S.; Menon, N. V.; Wang, Y.; Joshi, H.; Liu, S. Y.; Chong, K. C.; Kang, Y.; Sun, H. D.; Stuparu, M. C., All-organic luminescent nanodots from corannulene and cyclodextrin nano-assembly: continuous-flow synthesis, non-linear optical properties, and bio-imaging applications. *Mat. Chem. Front.* **2017,** *1* (5), 831-837.

34. Tian, Y. X.; Halle, J.; Wojdyr, M.; Sahoo, D.; Scheblykin, I. G., Quantitative measurement of fluorescence brightness of single molecules. *Methods Appl. Fluoresc.* **2014,** *2* (3), 035003.
